# Troubles With Tubules: How Do Iron‐Mineral Chemical Gardens Differ From Iron‐Mineralized Sheaths of Iron Oxidizing Bacteria?

**DOI:** 10.1111/gbi.70021

**Published:** 2025-05-14

**Authors:** Melanie Podbielski, Pamela Knoll, Georgia Brown, Sigrid Huld, Anna Neubeck, Julyan H. E. Cartwright, C. Ignacio Sainz‐Díaz, Carlos Pimentel, Sean McMahon

**Affiliations:** ^1^ Grant Institute, School of GeoSciences University of Edinburgh Edinburgh Scotland; ^2^ School of Biological Sciences University of Edinburgh Edinburgh Scotland; ^3^ School of Physics and Astronomy University of Edinburgh Edinburgh Scotland; ^4^ Department of Earth Sciences Uppsala University Uppsala Sweden; ^5^ Instituto Andaluz de Ciencias de la Tierra CSIC Granada Spain; ^6^ Instituto Carlos I de Física Teórica y Computacional Universidad de Granada Granada Spain; ^7^ Departamento de Mineralogía y Petrología, Facultad de Ciencias Geológicas Universidad Complutense de Madrid Madrid Spain

**Keywords:** biogenicity, dubiofossils, filaments, *Leptothrix*, microtubes, pseudofossils

## Abstract

Microscopic tubules and filaments composed of iron minerals occur in various rock types of all ages. Although typically lacking carbonaceous matter, many are reasonably interpreted as the remains of filamentous microorganisms coated with crystalline iron oxyhydroxides. Iron‐oxidizing bacteria (IOB) acquire such a coating naturally during life. However, recent debates about purported microfossils have highlighted the potential for self‐organized nonbiological mineral growth (particularly in chemical gardens) to form compositionally and morphologically similar tubules. How can biogenic and abiogenic iron‐mineral tubules be differentiated? Here, we use optical and electron microscopy and Mössbauer spectroscopy to compare the composition, microtexture, and morphology of ferruginous chemical gardens and iron‐mineralized sheaths of bacteria in the genus *Leptothrix*. Despite broad morphological similarity, we find that *Leptothrix* exhibits a narrower range of filament diameters and lower filament tortuosity than chemical gardens. Chemical gardens produced from a ferrous salt also tend to incorporate Fe^2+^ whereas *Leptothrix* sheaths predominantly do not. Finally, the oxyhydroxides formed in *Leptothrix* sheaths tend to be smoother and denser on the inward‐facing side, rougher and sparser on the outward side, whereas for chemical garden tubules the reverse is true. Some of these differences show promise for the diagnosis of natural samples.

## 1 Introduction

Astrobiologists and palaeobiologists have long struggled to distinguish between fossilized microbes and the products of non‐biological chemical activity. This question of “biogenicity” is particularly vexed with respect to the micrometer‐diameter hollow tubules and solid filaments of Fe‐, Mg, and Mn‐(oxhydr)oxides and clay minerals, which are found abundantly in agates, jaspers, cherts, and silicified or calcified cavities in basalts, limestones, and other rocks of all ages (Bengtson et al. 2017; Chi Fru et al. 2013; Hofmann and Farmer 2000; Little et al. 2004; Schumann et al. 2004; Trewin and Knoll 1999; Zhou et al. 2015; McMahon and Ivarsson 2019). These commonly occur as “filamentous fabrics”, in which individual filaments branch and interconnect to form dense networks reminiscent of modern microbial mats or fungal mycelia (Hofmann et al. 2008; Hofmann and Farmer 2000). Indeed, most of the geological filaments and tubules under consideration here have been interpreted as fossils of ancient bacteria or fungi. This interpretation is generally reasonable given that filamentous microbes are widespread on Earth, have an undisputed fossil record reaching back at least ~2.5 billion years (Javaux and Lepot 2018) and include iron‐oxidizing bacteria (IOB) such as *Leptothrix* whose sheaths can become biomineralized, coated with microcrystalline iron oxyhydroxides during life, essentially fossilizing themselves (Kunoh et al. 2015; van Veen et al. 1978). IOB are common in active hydrothermal systems where they form filamentous networks strongly resembling those found in the fossil record (Edwards et al. 2011; Little et al. 2004). Subfossil fungal hyphae partially encrusted with authigenic minerals have also been encountered in drill cores from the deep subsurface, consistent with the view that mineral filaments and tubes hosted in ancient cavities may provide a fossil record of Earth's “deep biosphere” (Drake et al. 2017; McMahon and Ivarsson 2019). Given that Mars is primarily a basaltic planet, the common occurrence of iron‐mineral tubules in mineralized basaltic cavities has also encouraged hopes that similar features might constitute biosignatures detectable by current or forthcoming rover missions (e.g., Onstott et al. 2019).

The genus *Leptothrix* is a widespread group of modern biomineralizing bacteria, found globally in streams and wetlands as well as deep wells, aquifers, and geothermal springs (Dondero 1975). All four *Leptothrix* species: *L. cholodnii, L* *. ochracea* , *L. lopholea*, and *L. discophora* , are filamentous and sheath forming with rod‐shaped cells. In nature, they form masses of biogenic iron‐oxide tubules, approximately 1 μm in diameter (Spring 2006). Cell diameters across all *Leptothrix* species are between 0.6 and 1.5 μm (Emerson and Ghiorse 1992; Spring 2006). Colonies sometimes grow large enough to obstruct drainage pipes and alter biogeochemical cycling in wetlands and water systems (Cullimore and McCann 1978). While commonly referred to as chemolithotrophic Fe(and Mn)‐oxidizers there is evidence that *Leptothrix* species may also metabolize sugars, polysaccharides, organic acids, or even sulfur species (Boogerd and De Vrind 1987; Spring 2006; Tothero et al. 2024). *Leptothrix* cells produce EPS sheaths which become coated in iron oxides. Negatively charged polymers found in the sheath play a role in binding iron to the sheath surface (Emerson and Ghiorse 1993; Suzuki et al. 2011). Though similar in overall morphology and habitat, individual *Leptothrix* species have some distinguishing features. *L. ochracea* is notable for rapidly forming large quantities of mineralized sheaths, containing very few cells; this species is also notoriously difficult to culture under laboratory conditions (Ghiorse 1984; Mulder 1964). *L. cholodnii* is grown more readily in the lab, with living cells more frequently observed inside sheaths (Mulder and Van Veen 1963). Biomineralizing filamentous bacteria similar to *Leptothrix* (perhaps even ancestral strains) have a greater preservation potential in the rock record compared to other microbes due to their prodigious production of robust mineralized sheaths, and are therefore of interest to paleo‐ and astrobiologists (Hirsch et al. 2025; Picard et al. 2015).

On the other hand, there is a concern that some of the ferruginous tubules and filaments in the rock record could be the result of non‐biological physico‐chemical processes; most lack organic carbon (McMahon and Ivarsson 2019). Nature produces abundant filaments abiotically, from acicular crystals and linear crystalline aggregates to volcanic glass fibres. Isopachous coating or diffusive boundary migration can turn an originally linear object into a tube with a circular cross section (as commonly seen in agates). More generally, the simple morphology of many purported microbial fossils is of limited value as a biosignature in itself, since it can be mimicked by a range of life‐like structures that have nothing to do with life (Brasier et al. 2002; García Ruiz et al. 2002; Hofmann 1972; McMahon 2019; Nims et al. 2021; McMahon and Cosmidis 2022). The origin of the filamentous fabrics in “moss agates” has been debated inconclusively for more than two centuries (Bowerbank 1842; Brown 1957; Daubenton 1782; Göppert 1848; Götze et al. 2020; Hofmann and Farmer 2000; Liesegang 1914; Mac Culloch 1814; McMahon 2019). Hopkinson et al. (1998) argued that iron–mineral filament networks around hydrothermal vents form by a self‐organized diffusion–reaction‐precipitation process within a preexisting mass of silica gel subject to strong chemical gradients. Other workers have suggested that many similar tubules and filaments may result from natural *chemical gardens* (Hawley 1926; Brown 1957; McMahon 2019; Johannessen et al. 2019). In the famous benchtop experiment first reported by Glauber (1646), chemical gardens “grow” from reactions between dissolved or dissolving transition metal salts and alkaline aqueous solutions. A gelatinous, semipermeable, largely siliceous membrane forms in seconds‐to‐minutes at the interface between the acidic transition‐metal salt solution (e.g., of iron sulfate) and the alkaline medium. An increase in osmotic pressure causes this membrane to rupture and expel fine jets of fluid that are simultaneously enclosed by newly‐formed, tubular extensions of the membrane. These harden over hours‐to‐days into brittle, branching tubules (with an outer layer of silica encrusted by an inner layer of, for example, iron oxyhydroxide; Kotopoulou et al. 2021) with lifelike trajectories and nearly perfect circular cross‐sections. The reaction is experimentally facile and can easily be used to produce iron oxide tubules in the laboratory that closely resemble the arrangement, morphology, and composition of many of those found in the rock record (Knoll et al. 2022; McMahon 2019; McMahon et al. 2021).

Chemical gardens are expected to form in nature; hydrothermal vent chimneys can be considered large‐scale examples, although these precipitate from reactions between aqueous solutions and not from the initial dissolution of “seed” grains (Barge et al. 2015a, 2015b). The hematite filaments and tubules reported by Dodd et al. (2017) from Archean hydrothermal cherts in Arctic Canada, initially described as Earth's oldest fossils (but see Lan et al. (2022) for reevaluation of the age and context), are especially similar to laboratory‐grown chemical gardens (McMahon 2019), as are hematite+talc filaments reported by McMahon et al. (2021) from a Jurassic subseafloor serpentinite deposit exposed in the Ligurian ophiolite of Northwest Italy. Similar tubules found as corrosion rusticles on iron shipwrecks have also been considered analogous to chemical gardens (Silva‐Bedoya et al. 2021). However, in all these cases (except for hydrothermal vent chimneys), the possibility of a biological origin cannot be excluded. There is now a clear need for improved criteria to differentiate between chemical gardens and the fossil remains of filamentous microorganisms, particularly where there is too little carbonaceous residue for the detection of biomolecules (McMahon 2019; Johannessen et al. 2019).

Here, we report the results of laboratory experiments designed to identify disambiguating characteristics in IOB and iron chemical gardens. We cultured IOB colonies of the common genus *Leptothrix* and compared their biomineralized sheaths with the iron oxyhydroxide chemical gardens produced by adding iron (II) sulfate heptahydrate to a sodium silicate solution. Scanning electron microscopy (SEM), transmission electron microscopy (TEM), Mössbauer spectroscopy, and optical microscopy and morphometry were used to characterize the experimental products.

## 2 Methods

### 2.1 (a) Leptothrix: Morphology and Culturing

*Leptothrix* bacteria were obtained from two sources. Firstly, *Leptothrix cholodnii* strain SP‐6 was purchased from ATCC (catalog #51168). Secondly, wild *Leptothrix* sp. colonies were cultured from a ~ 10 mL sample collected in a sterile 15 mL Falcon tube from rusty, slightly iridescent boggy ground (Schmidt et al. 2014) near Figgate Burn, Edinburgh, UK (55°56′54.5″N 3°07′56.1″W). Approximately 1 mL of this environmental sample was pipetted into 100 mL glass bottles containing ~75 mL of a sterile nutrient‐poor liquid medium used by Angelova et al. (2015) for isolating *Leptothrix*, with sterilized iron filings as an Fe source After cultivation at 25°C in the dark (to suppress cyanobacteria) for 8 days, sheathed filamentous bacterial colonies proliferated and were morphologically identified as a species of *Leptothrix*. Hereafter we refer to the latter strain as “*Leptothrix* sp. (FB)”. No differences were apparent at any stage between these two populations. All *Leptothrix* cultures were grown in unsealed 100 mL bottles containing 75 mL Angelova isolation medium (I.M.) (Angelova et al. 2015) (see Appendix S1) at ambient temperature in the dark and subcultured after 1 week of growth.

### 2.2 (b) Chemical Garden Experiments

Chemical gardens were produced following the method of McMahon (2019) by manually dispersing crystalline granules (seed grains) of iron(II) sulfate heptahydrate (98% FeSO_4_.7H_2_O; Alfa Aesar, Heysham, UK) into 15 mL aliquots of either sodium silicate solution or sodium carbonate solution. Seed grains were powdered by mortar and pestle and sieved to < 63 μm. Sodium silicate solution was prepared using ultrapure deionized water (Thermo Scientific Barnstead Nanopure) and 100 g L^−1^ of sodium silicate powder (53% SiO_2_, 26% Na_2_O, Scientific Laboratory Supplies, Nottingham, UK). Sodium carbonate solutions were prepared using sodium carbonate monohydrate powder (reagent grade, 124 g/mol Na_2_CO_3_.H_2_O; Emprove Essential, Merck). Filaments were rinsed in deionized water prior to analyses. For one experiment, the filaments were then placed in the I.M. at ambient pressure and temperature (≈ 20°C) with no light exposure and observed microscopically after 21 days.

### 2.3 (c) Geological Samples

A petrographic thin section of Devonian basalt‐hosted moss agate from Campsie, Scotland (GPS: 55.98, −4.20) was prepared from a chip (Naturhistorisches Museum Bern, catalog number 31995) kindly provided by B. Hofmann. The same material was studied by Hofmann et al. (2008). It was processed into thin sections and examined with an optical microscope (Figure S1).

A hand‐sample of calcite‐veined lacustrine limestone from the Devonian deposits near Fochabers, NE Scotland (the Tynet Burn fish bed) was generously provided by J. Parnell from the museum collection of the School of Geosciences in the University of Aberdeen; this sample represents the same material studied by Trewin and Knoll (1999). It was processed into thin sections and examined with a Zeiss SUPRA 35VP with an RBSR BSE detector. The accelerating voltage was 4 kV, and the working distance was 8.5 mm (Figure S2a,b).

A hand‐sample of iron‐stone comprising densely packed tubules of goethite was collected in the field from the Cerro Colorado zone in old palaeoterraces (GPS: 37.70919, −6.59377) close to the Alto de la Mesa area in the village of Minas de Riotinto in Huelva province of Andalusia region, Spain. This material represents fossilized Rio Tinto river terrace deposits about two million years old (Fernández‐Remolar and Knoll 2008). It is equivalent to the material studied by Barge et al. (2016). It was examined with an FEI Quanta 400 scanning electron microscope (SEM) at the University of Granada, with a variable working distance (7–12 mm) and an accelerating voltage of 5 kV (using the Everhart–Thornley detector) or 20 kV (using the concentric back‐scattered detector).

### 2.4 (d) Microscopy and Morphometry

Transmitted light microscopy of microbial samples and chemical gardens was conducted on a Leica DM 4000 B microscope equipped with 5, 10, 20, 40, and 100× objectives, and a DFC 450C camera. Some lower magnification observations were made on a Leica Zoom 2000 microscope and photographed using a mobile phone camera (Galaxy S10e). Observations of geological samples in thin‐section were made on a Leica DM2700 P reflected/transmitted light polarizing microscope with a DFC 420C camera. Images were acquired using Leica Application Suite v 4.0.

For observation using scanning electron microscopy, selected experimental samples were placed on stubs and allowed to air dry for at least 2 h in a laminar flow hood. Specimens were either gold‐coated or left uncoated and were examined using a Zeiss Crossbeam 550 FIB‐SEM operated at an accelerating voltage of 5 kV and a probe current of 100 pA. Secondary electron signals were detected using an InLens detector. For transmission electron microscopy, experimental samples were fixed, cut into ultrathin sections, stained, and viewed in a JEOL JEM‐1400 Plus TEM (see Appendix S1).

Morphometric analysis was carried out using the methods of Hofmann et al. (2008). Filaments were identified in optical photomicrographs. Each filament was traced and divided into straight segments of uniform length. The *x,y* coordinates of each end of the filament and of the connection points of the segments were obtained using the ImageJ (Schneider et al. 2012) software, with the coordinates recorded in μm using the scale‐bars for calibration. These coordinates were then used to calculate the tortuosity (sum of segment lengths divided by the length of a straight line from beginning to end of each filament, that is, a straight line drawn from the first *x,y* coordinate to the last) and bending (average magnitude of inclination of each segment compared to the preceding segment) parameters. For a perfectly straight filament, tortuosity would be 1 and bending would be 0°/μm.

### 2.5 (e) Mössbauer Spectroscopy

Iron oxidation state(s) in the experimental samples were analysed using Mössbauer spectroscopy at the Swedish Museum of Natural History in Stockholm using a custom‐built spectrometer system operated in constant acceleration mode. Powdered samples (10 mg) of tubes from 16‐day old *Leptothrix* SP‐6 cultures, chemical gardens formed in a carbonate solution (30 mg), and chemical gardens formed in a silicate solution (34 mg) were mixed with Mössbauer absorbers, mixed with an acrylic resin and then pressed, under mild heating, into 12‐mm‐diameter discs. The spectra were collected at room temperature using a standard Co‐57 source in a Rh matrix with a nominal activity of 50 mCi. Spectra were acquired over 1024 channels in the velocity range − 4.5 to +4.5 mm/s and calibrated against an α‐Fe foil before folding. The least‐squares fitting software MossA 1.01f (Prescher et al. 2012) was applied to analyse the obtained spectra using a fitting model with one doublet assigned to Fe^3+^ and three doublets to Fe^2+^ (Lenaz et al. 2018).

### 2.6 (f) X‐Ray Diffraction Analysis

Single‐crystal X‐ray diffraction data were collected using a Bruker D8 Venture diffractometer equipped with Mo Kα radiation (*λ* = 0.71073 Å) generated at 50 W (50 kV, 1 mA). The data were acquired at room temperature with an exposure time of 60 s per frame. Powder X‐ray diffraction data were collected using a Rigaku MiniFlex diffractometer. Samples were manually ground in an agate mortar before analysis. Data were recorded over a 2θ range of 2° to 70° with a step size of 0.01° and a scan speed of 1° per minute. Diffraction patterns were analysed using MDI Jade software with the ICDD PDF database for phase identification.

## 3 Results

### 3.1 (a) Qualitative Microscopy: Leptothrix and Chemical Gardens

No differences in morphology or mineralization behavior were apparent between *Leptothrix cholodnii* SP‐6 and the *Leptothrix* sp. (FB) we obtained from the wild. The sheathed filaments exhibited varying degrees of mineralization and encrustation (Figure S3). Overall, sheaths varied from semitransparent to yellowish to deep reddish brown. Less mineralized “immature” filaments were smoother, less opaque and tended to be highly sinuous with relatively few breaks or sharp bends when observed in growth medium on wet‐mount slides (Figure S3e) (see Section 3.3). Once removed from liquid culture and dried for SEM observation “immature” sheaths became more brittle and prone to breakage; this was also noted by Vesenka et al. (2018). More “mature” sheaths appeared increasingly opaque, with a rusty orange to brown coloration due to the deposition of iron oxides, and their smooth sheath surfaces became loosely blanketed by flocculent deposits (Figure S3c,d,f). The most mature sheaths were typically observed to be less curved (see Section 4.4). They also tended to be more brittle while still in water or media, a trend also observed by Van Veen and others (van Veen et al. 1978; Spring 2006). Highly mineralized filaments were sometimes observed to coalesce into a single multifilament structure (Figure S7). Some larger examples of these were over 1 mm in length and heavily encrusted (Figure S4).

**FIGURE 1:**
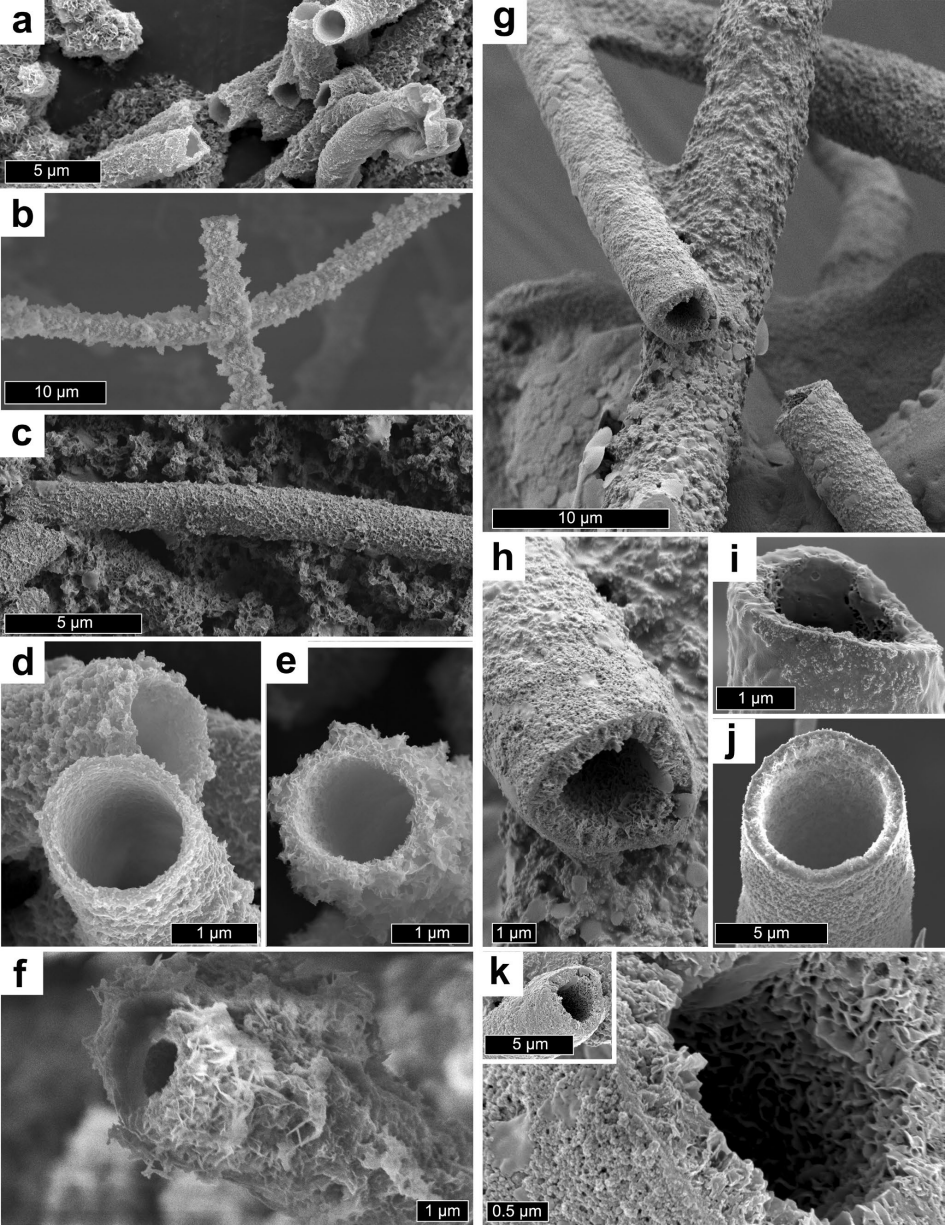
Scanning electron micrographs of iron‐mineral tubes produced by *Leptothrix cholodnii* SP‐6 (a, c, d), *Leptothrix* sp. (FB) (b, e, f), and chemical gardens (g–k). *Leptothrix* filaments are smooth on their inner surfaces and rougher on their external surfaces (a–c). Chemical garden tubules have relatively smoother outer surfaces and more diffuse and variable inner surface textures (d–f).

Tubular *Leptothrix* sheaths were observed using scanning electron microscopy (SEM). Immature sheath surfaces appeared mostly smooth and semitransparent (Figure S5) whereas more mature *Leptothrix* tubules tended to have a smooth inner surface and a more irregular and roughly textured outer surface (Figure 1a–f). The tubules were consistently ~1 μm in diameter (Figure 1a–f). *Leptothrix* samples observed with SEM were not chemically fixed, and no preserved cell material was observed.

Chemical garden tubules formed by the reaction of iron (II) sulfate particles with a sodium silicate solution were consistently larger than the *Leptothrix* sheaths (see Section 3.2) but superficially similar. SEM observations of the chemical gardens revealed two main surface textures. The inner surfaces of the tubes were less dense, with a highly porous platy crystalline texture. The outer surfaces were denser and smoother (Figure 1g–k).

**FIGURE 2:**
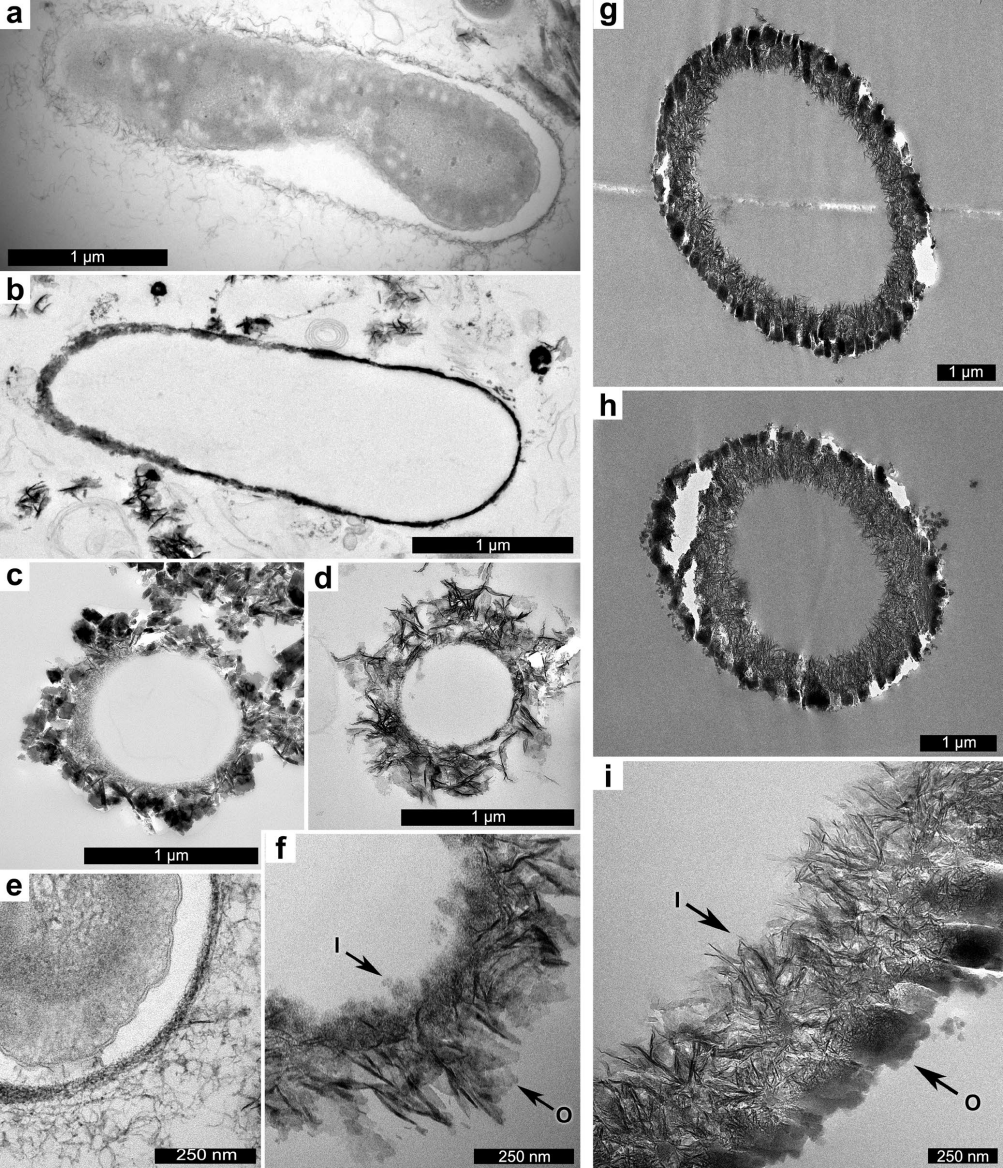
Transmitted electron micrographs of *Leptothrix cholodnii* strain SP‐6 (a, c, d), Leptothrix sp. FB (b, e, f), and chemical garden tubules (g–i). Biogenic filaments have well constrained tubule diameters (a–f) whereas chemical garden tubules have more variable diameters (g–i). Rough and diffuse textures are observed on the exterior surfaces of biogenic tubules with smoother inner surfaces (a–f). Chemical garden tubules have smoother outer surfaces and more diffuse and variable inner surface textures (g–i). White areas are due to sample charging (g, h). At higher magnification the Leptothrix sheath (f) is platy and irregular on the outside surface (O) and relatively smooth, fine and dense on the interior surface (I); whereas the chemical garden tube (i) is platy and irregular on the inner surface texture (I) and relatively smooth, fine and dense on the outer surface (O).

Younger (8 day old) and older (93 day old) *Leptothrix* sp. (FB) cultures were observed using transmission electron microscopy (TEM) (Figure 2a–f, Figure S6, & Figure S7). Examples from the 8 day old culture (Figure 2a,b,e) had relatively thin mineralized sheaths. Intact cells were occasionally revealed within the sheaths of bacteria from the 8 day old culture, although these were usually not present in the narrow plane of the ultrathin section. Examples from the 93 day old culture contained highly mineralized sheath exteriors (Figure 2c,d,f) displaying diverse granular (Figure 2c) platy (Figure 2d), and intermediate textures. Material coating the inner surface of the *Leptothrix* sheaths was typically smooth and fine‐grained compared to the platy and irregular outer surface. The outer surface of the chemical garden tubules appeared dark and dense in TEM micrographs (Figure 2g–i and Figure S6e,f), with some electrical charging and tearing (in white) apparent in these areas. This microtextural difference between *Leptothrix* and chemical gardens was especially clear in higher magnification TEM micrographs (Figure 2f,i).

### 3.2 (b) Quantitative Morphometry: Leptothrix and Chemical Gardens

**FIGURE 3:**
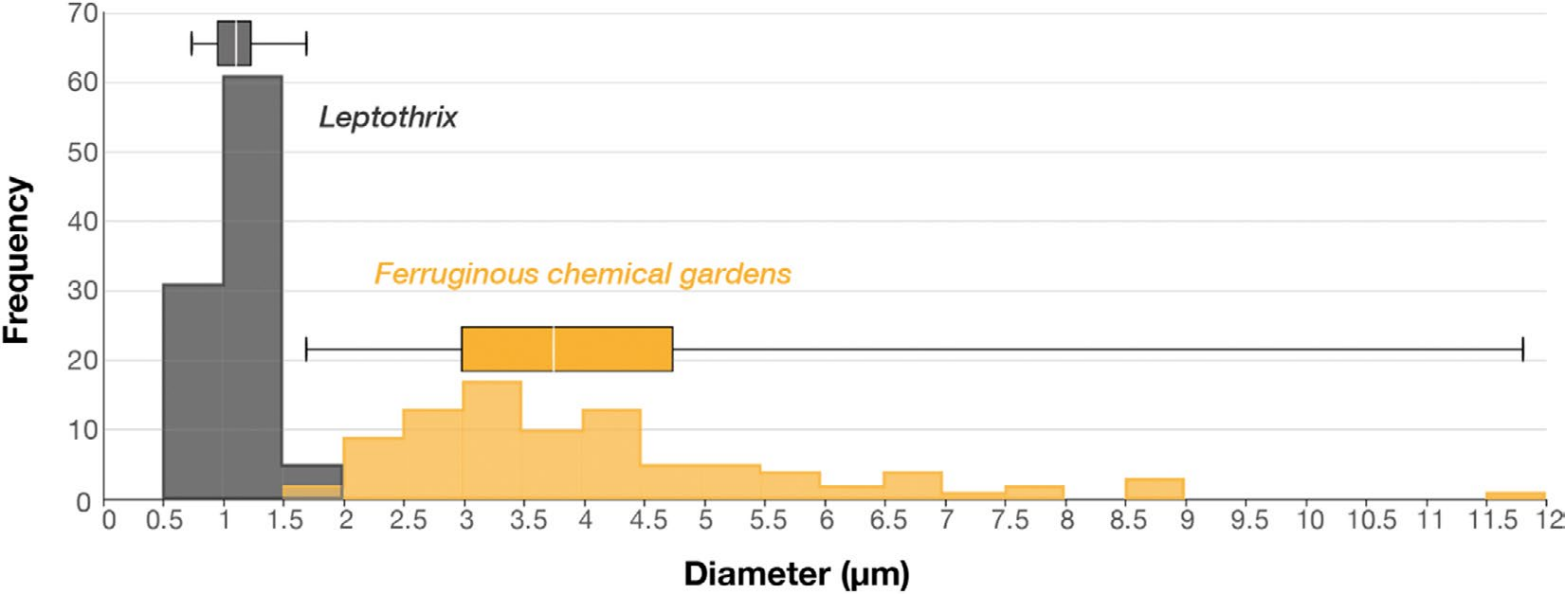
External diameters of Leptothrix sp. (FB) and ferruginous chemical garden tubes analysed in this study (histogram and box‐and‐whisker plots). Leptothrix cultures analysed were between 0 and 36 days old; chemical gardens were observed ~24 h after formation. It should be noted that smaller chemical garden tubes and apparently wider Leptothrix filaments are reported in other studies (see Section 4).

Leptothrix sheaths differed from the ferruginous chemical garden tubes produced in this study in their internal and external diameters (Figure 3; Table S1). The “mature” *Leptothrix* sheaths were no more than 1.7 μm in external diameter, with a median of 1.1 μm (*n* = 98). In TEM images, mineralized sheath wall thickness was typically between 20 and 120 nm (as seen in Figure 2a–f). The largest measured external diameter of a *Leptothrix* filament was about equal to the smallest measured diameter of a chemical garden tube (1.7 μm). However, chemical garden tubes showed a much broader distribution of external diameters, ranging up to 11.8 μm with a median of 3.7 μm. Additionally, older *Leptothrix* sheaths tended to acquire a loose coating of irregular masses of iron (oxyhydr)oxides, up to a thickness of at least 150 nm. If these are included in the measurement of tube diameter, then many are around 3 μm and some exceed 5 μm. This did not occur in chemical garden experiments because iron (oxyhydr)oxides deposit predominantly on the inward‐facing walls of (initially largely siliceous) chemical garden tubes, not the outer walls (Kotopoulou et al. 2021). This is because iron is supplied to the inside of the tubes (from the dissolving seed grains) and not the outside (the chemical gardens were not produced in an Fe‐rich liquid medium).

**FIGURE 4:**
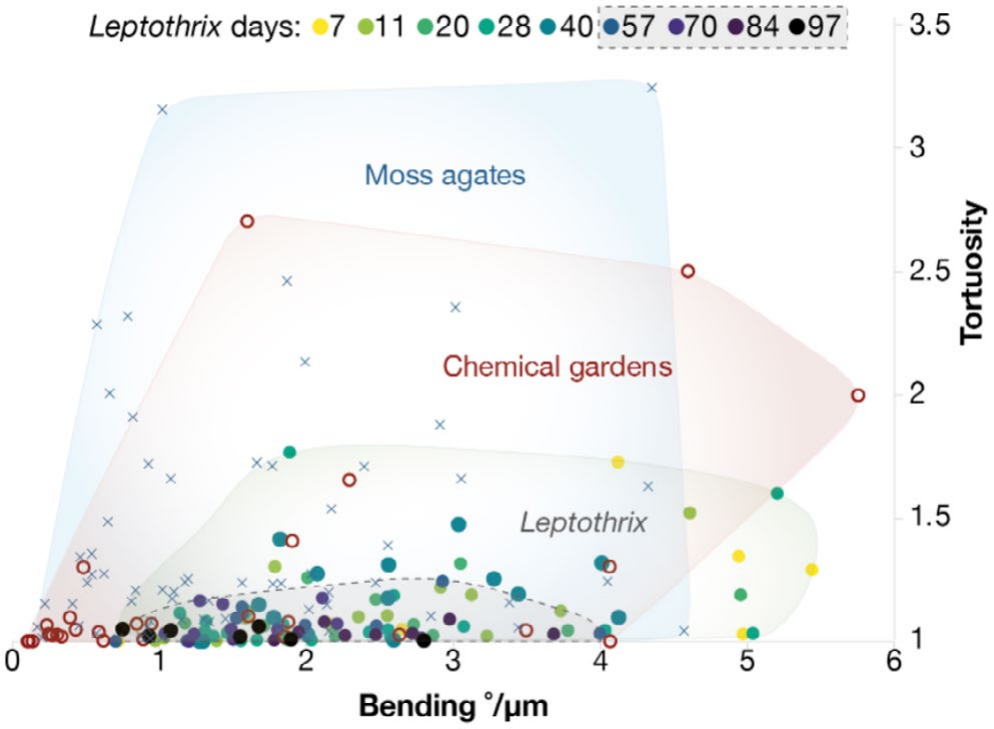
Morphometry of individually measured tubes/filaments. Tortuosity and bending do not discriminate between Leptothrix (filled circles, *n* = 106), chemical gardens (open circles, *n* = 30), or moss agate filaments (×, *n* = 67). However, the moss agate and chemical gardens show a wider range of tortuosity values than Leptothrix. Note Leptothrix bunching towards lower bending and tortuosity values over time (dashed line encloses Leptothrix cultures at least 57 days after inoculation).

Chemical garden tubes and *Leptothrix* sheaths were similar in bending (°/μm) and tortuosity values, although *Leptothrix* occupied a narrower range (Table S1; Figure 4). Bending and tortuosity decreased over time in *Leptothrix* cultures as the filaments became more mineralized, straighter, and more rigid on average (Figure S8). Fourteen *Leptothrix cholodnii* SP‐6 filaments sampled and measured 11 days after inoculation in fresh medium ranged in tortuosity from 1.00 to 1.52 (median = 1.10) and 0.97 to 4.61°/μm in bending (median = 2.26°/μm); fifteen filaments sampled on days 84 and 97 ranged from 1.00 to 1.09 in tortuosity (median = 1.03) and from 0.75° to 3.68°/μm in bending (median = 1.95°/μm); the overall decrease and range‐narrowing in both parameters is evident in both Figure 4 and Figure S8. Chemical gardens and filaments from a moss agate were similarly distributed in morphological parameter space (“morphospace”), and both reached much higher maximum values of tortuosity than the *Leptothrix*. For chemical gardens (*n* = 30), bending ranged from 0.10 to 5.75 (median = 0.79) and tortuosity ranged from 0.97 to 2.71 (media*n* = 1.04). For the moss agate filaments (*n* = 69), bending ranged from 0.17 to 12.2 (median = 1.3) and tortuosity ranged from 1.01 to 3.24 (median = 1.22). (In a similar sample from the same locality, Hofmann et al. (2008) reported average bending and tortuosity values of 1.84 and 1.11 respectively). By contrast, the highest tortuosity measured for *Leptothrix* was 1.80 (on day 28).

### 3.3 (c) Mössbauer Spectroscopy: Leptothrix and Chemical Gardens

**FIGURE 5:**
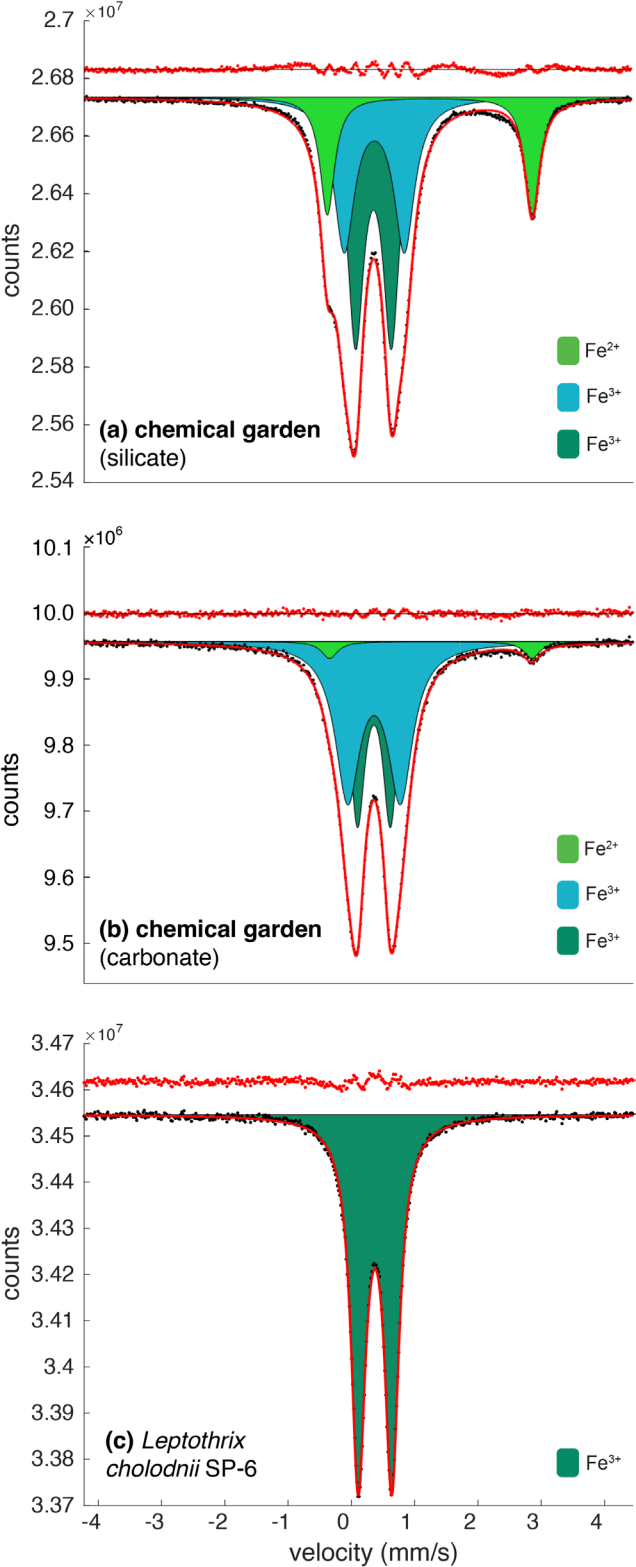
Deconvolved Mössbauer spectra for *Leptothrix cholodnii* SP‐6, chemical gardens prepared in carbonate solution and chemical gardens prepared in silicate solutions. The spectral components (doublets) are shown in different colors (the dark green and teal components are assigned to Fe^3+^; light green Fe^2+^) and described in Table S2. In the chemical gardens (a, b), Fe^2+^ is observed and the Fe^3+^ is quite broad reflecting the environment; whereas the *Leptothrix* sample (c) shows a single doublet indicating that only Fe^3+^ is present, and its environment is homogeneous.

Doublet peaks corresponding to Fe^3+^ and Fe^2+^ are evident in the Mössbauer spectroscopic analysis (Figure 5), showing clear differences in Fe^2+^/Fe_tot_ values between the samples, with 0% Fe^2+^/Fe_tot_ in the *Leptothrix* tubes, 4.1% in the chemical garden tubes prepared in carbonate solution and 19.8% in the chemical garden tubes prepared in silicate solution (Table S2). The error is estimated to be approximately 2%.

### 3.4 (d) X‐Ray Diffraction Analysis

Single‐crystal X‐ray diffraction analysis was attempted to determine the crystallographic structure of both chemical garden and biomineralized sheath material. However, the collected diffraction data showed only diffuse scattering, indicating that the material is amorphous or poorly crystalline. A powder X ray diffraction pattern with some visible peaks was obtained for the chemical garden sample. The pattern was predominantly amorphous, displaying broad, diffuse features, but a few distinct peaks were observed, suggesting the presence of a minor crystalline phase. These peaks are consistent with the diffraction pattern of the ferric oxhydroxide mineral, ferrihydrite, though other phases may also be present (Figure S9).

### 3.5 (e) Qualitative Microscopy: Geological Samples

**FIGURE 6:**
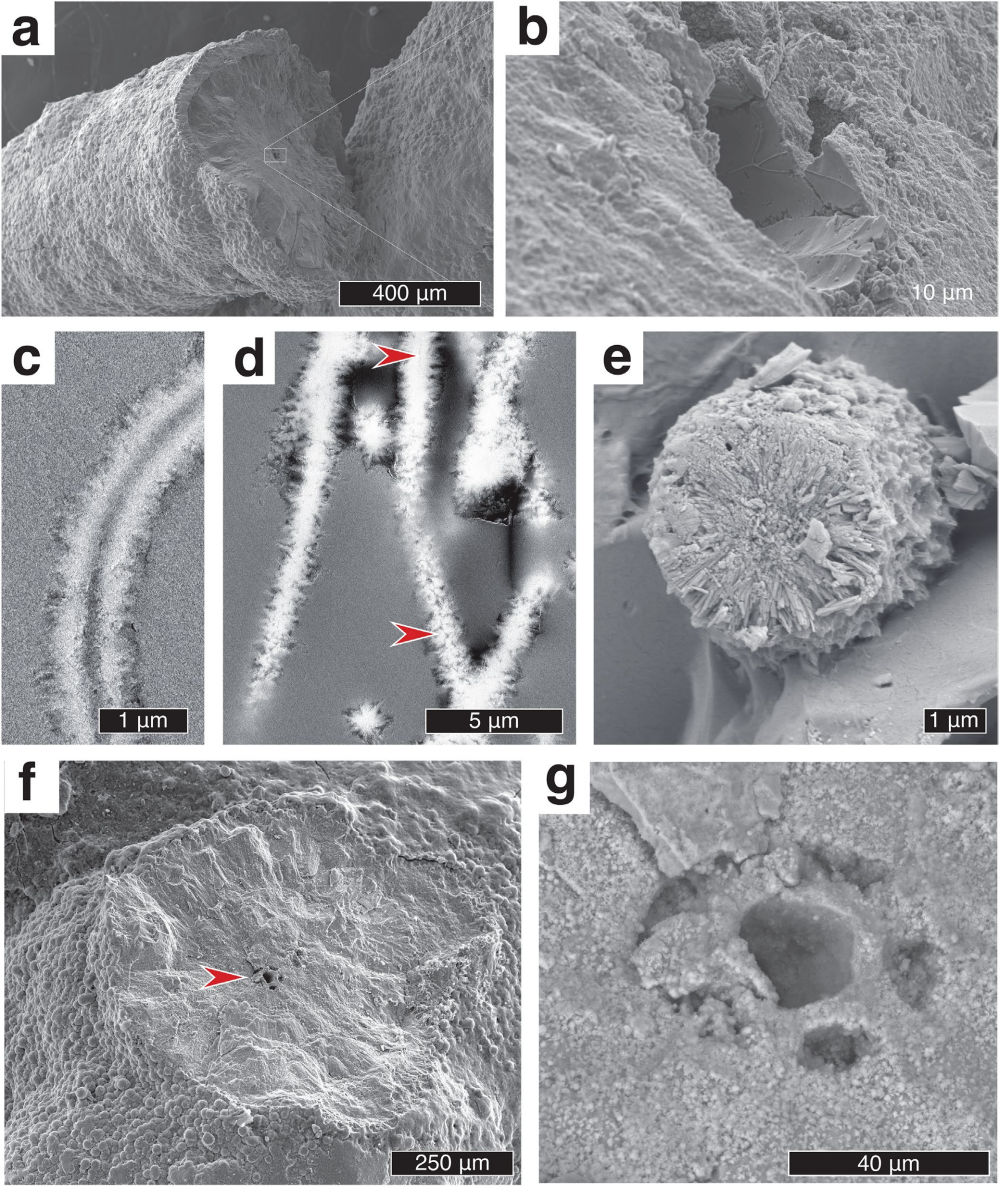
Scanning electron micrographs showing microtextural features of naturally occurring iron (oxyhydr)oxide tubules from a fluvial ironstone ~ 2 million years old cropping out at Cerro Colorado, Spain (a, b) and from a lacustrine limestone ~ 390 million years old cropping out near Fochabers, NE Scotland (c–e). (a) Rough‐textured exterior typical of Cerro Colorado filaments. (b) Interior of the filament bears the smooth imprint of the plant tissue on which the iron oxide precipitated. (c) Cross‐section shows relatively smooth inner surface and relatively coarse outer surface. (d) Cross section showing occlusion of the interior space by recrystallization: Where the interior cavity is still visible, it is smooth in some places (upper arrow) but irregularly sutured in others (lower arrow). (e) Filaments are composed of radially disposed thin crystallites. Here no central cavity is visible. (f) An extensively mineralized Cerro Colorado filament is observed with the interior cavity still visible (arrow). (g) At higher magnification, the petaloid cavity cross‐section can be observed.

Microtextural features of naturally occurring iron (oxyhydr)oxide tubules from a fluvial ironstone ~2 million years old cropping out at Cerro Colorado, Spain, and from a lacustrine limestone ~390 million years old cropping out near Fochabers, NE Scotland, were observed using scanning electron microscopy (Figure 6).

## 4 Discussion

The mineralized *Leptothrix* sheaths and abiotic chemical garden tubules produced in this study are morphologically and chemically similar in many ways. However, our results show that these structures can be differentiated on both textural and chemical grounds, at least prior to diagenesis or other processes of alteration (see Section 4.5 for a discussion of the preservation potential of these differences).

### 4.1 (a) Microtexture Differentiates Leptothrix Sheaths and Chemical Garden Tubes

*Leptothrix* sheaths and chemical garden filaments show a variety of surface microtextures, ranging from a smooth, dense wall to a loose, porous mesh of platy crystals. In our experiments, *Leptothrix* tubes typically showed smooth and dense interior layers and coarser, more porous outer layers, with variably platy or blocky crystal morphologies. *Leptothrix* tubules with smooth outer surfaces were sometimes observed in young (< 10 day old) cultures, but the outer surfaces rapidly accumulated secondary mineralization, resulting in varied rough textures; smooth exterior sheaths were rarely observed in cultures over 10 days old. The denser inner layer and a more diffuse outer layer of *Leptothrix* sheaths have been noted by other researchers, though the inner denser layer was interpreted by Suzuki et al. as forming secondarily to the outer diffuse layer (Suzuki et al. 2011; Kunoh et al. 2016b). This distinctive difference between inner and outer surface textures was observed by Suzuki et al. (2012) in cultures grown in naturally iron‐rich groundwater and also in cultures grown in culture medium with iron plates, indicating this trend is consistent whether the iron source is Fe^2+^or Fe^0^.

The extensive mineralization observed on sheath exteriors (and occasionally interiors) in old (93 day) *Leptothrix* cultures is almost certainly independent of active cell metabolism. These oldest cultures showed no signs of active growth, and even young (< 10 day old) cultures observed with fluorescence microscopy (see Appendix S1 section) contained very few living cells (Figure S10). However, iron oxides still accumulate over time due to continued sheath surface mineralization independent of biological activity (Kunoh et al. 2016a). Past studies have shown that media composition can have a significant effect on *Leptothrix* sheath mineralization and specifically on iron speciation (Angelova et al. 2015; Shopska et al. 2017). Samples taken from naturally occurring iron‐oxidizing bacteria communities also demonstrate that even small differences in the growth environment can result in biogenic iron deposits with distinct iron species ratios (Gualt et al. 2011; Tuhela et al. 1997). Further work on the biomineralization and taphonomy of *Leptothrix* sheaths is needed to more fully understand the effects of specific environmental factors including temperature, pH, dissolved mineral concentrations, and dissolved oxygen levels on sheath formation, filament morphologies, and mineral speciation.

Chemical garden tubes showed the reverse: external surfaces were smooth, while inner surfaces were coarser, more diffuse, and more variable. It has been noted that chemical garden filament wall thickness increases only in an inward direction during the primary growth stage, with the accumulation of metal oxyhydroxides (Roszol and Steinbock 2011; Wang et al. 2017). By contrast, mineralization of *Leptothrix* sheaths is predominantly on the outer surface, mediated by exopolymer fibrils on the outside of the sheath (Kunoh et al. 2017). More generally, we would suggest that any mineral coating precipitated on the outer surface of any organism is likely to show a smooth, fine interior wall (since its growth initiates on and is and bounded by the smooth biological substrate, with which it must be conformable) and a relatively coarse and irregular exterior texture (since there is no such constraint). Similarly, the outer surface of the iron (oxyhdr)oxide layer of the chemical garden tubes must be relatively smooth because it conforms with the smooth siliceous membrane on which it forms, while the inside surface can be arbitrarily irregular. Even after *Leptothrix* cells have abandoned the tubules or died and lysed, the interior surfaces of the tubules show reduced (abiotic) mineralization compared to their outer surfaces. This may be due to the inner surfaces being less exposed to mineral‐rich fluids or because these two surfaces have distinct and different affinities for binding minerals.

### 4.2 (b) Redox State Differentiates Leptothrix Sheaths and Chemical Garden Tubes

In our experiments, chemical garden tubes contained Fe^2+^ and *Leptothrix* sheaths did not. *Leptothrix* oxidizes Fe^2+^ into Fe^3+^ metabolically, while Fe^2+^ oxidation and deposition of Fe^3+^ also take place independent of cell metabolism (Kunoh et al. 2016b). Both processes explain the lack of Fe^2+^ in the precipitates of *Leptothrix* sheaths. Other studies have also noted the absence of Fe^2+^ in mineralized *Leptothrix* sheaths, with Ferrihydrite, Lepidocrocite, and Goethite predominating (Kunoh et al. 2016b; Shopska et al. 2017; ThomasArrigo et al. 2022), though magnetite has also been detected in some *Leptothrix* biofilms (Angelova et al. 2015; Nedkov et al. 2016).

The formation of iron‐rich chemical gardens is less straightforward; iron oxidation is not an integral part of chemical garden formation but rather an unintended consequence of performing the experiment in an aerobic environment. If the experiment had been performed anaerobically and the precipitates analysed without being exposed to oxygen, the measured balance of Fe^2+^ and Fe^3+^ would likely have reflected that of the seed salt material (Barge et al. 2016). However, the difference in Fe redox state between the silicate and carbonate experiments in this study implies an effect of the anion. Silica is incorporated into the walls of chemical gardens formed in silicate solutions (Barge et al. 2015a, 2015b) and likely reduces the exchange of Fe^2+^ and Fe^3+^ while forming an impermeable barrier to air, thereby slowing the oxidation of Fe^2+^ (Piispanen and Sallanko 2011; Wolthoorn et al. 2004). In carbonate‐rich environments, carbonate ions (CO_3_^2−^) can stabilize Fe^2+^ ions by forming complexes such as FeCO_3_ or Fe(CO_3_)_2_^2−^, reducing their tendency to oxidize by keeping them in a dissolved state (Musić et al. 2004). However, in high carbonate concentrations, soluble iron‐carbonate complexes may form, allowing Fe^2+^ to remain reactive and eventually oxidize to Fe^3+^ under favorable conditions. This dual role of carbonate, initially stabilizing Fe^2+^ while also enabling oxidation under specific conditions, reflects its complex influence on iron redox chemistry in natural systems.

### 4.3 (c) Size Distribution May Possibly Differentiate Leptothrix Sheaths and Chemical Garden Tubes

The populations of tubules we observed were obviously different in size; all *Leptothrix* sheaths were ≤ 1.7 μm in diameter whereas all chemical garden tubules were ≥ 1.7 μm. The degree of variability in filament diameter was also distinct between the two groups. *Leptothrix* filaments vary with a standard deviation of 0.20, whereas chemical garden filaments vary with a standard deviation of 1.82.

Fe‐oxide encrusted microbial filaments in microbial mats at modern hydrothermal vent sites are observed to have similar filament diameters to those recorded in our work (Emerson and Moyer 2002; Fleming et al. 2013; Scott et al. 2015). In a study of iron oxyhydroxide filaments occurring in siliceous hydrothermal mounds and chimney structures from the Arctic Mid‐Ocean Ridge, Johannessen et al. (2019) observed several different populations, with median diameters of 1.7, 3.3, 3.4, 8.8, 10.7, and 24.25 μm, and considered (on the basis of several lines of evidence) that the filaments with larger diameters were more likely to be abiotic. Our results tend to support this conclusion. However, caution is needed when attempting to diagnose the biogenicity of natural filaments or tubules on the basis of diameter (especially external diameter). Other experiments have produced chemical garden tubules with consistent diameters < 1 μm (albeit using Mn rather than Fe; Huld et al. 2023), and *Leptothrix* sheaths can accumulate flocculent coatings of at least 10 μm thickness (see Figure 4D in Schmidt et al. 2014). Chemical garden tube diameters are variable since tube diameter varies according to fluid flow. In a chemical garden, the tube grows around a jet of fluid that acts as a template. In principle, a chemical garden tube radius could be arbitrarily small or large were the flow rate to be arbitrarily small or large. Physical modeling of this process (Cardoso and Cartwright 2017) shows that tube radius varies with flow rate, following Poiseuille flow driven by a pressure gradient, −dP/dz., and a density difference giving buoyancy forces Δρg. The density difference is fixed by the chemical species involved. In some cases, the pressure gradient may come from outside forcing, as when there are external fluid flows. But in the instances we are discussing here, without any external flows, the pressure gradient comes from osmotic forces within the system, which are again fixed by the concentrations of chemical species inside and outside a chemical garden.

Putting all this together, the implication is that the chemical garden tubes in the small geological systems we are discussing have a range of diameters limited by the maximum and minimum osmotic and buoyant forces in a self‐organized system. In our experiments, grains were constrained to < 63 μm diameter, resulting in a distribution of chemical garden tube diameters very similar to that reported by McMahon (2019) from experiments using the same reagents and conditions. A greater variability in seed grain size would induce a greater variability in chemical garden tubule diameter.

Filaments that vary over an order of magnitude in their external diameter might represent chemical gardens, but could also simply represent a mixed population of organisms or a variably encrusted assemblage of originally equidimensional filaments (Provencio et al. 2001). Similarly, filaments with highly constrained diameters might represent fossils of the same taxon (such as *Leptothrix*), abiotic mineral fibres (Hofmann et al. 2008; Muscente et al. 2018) or other non‐biological products, including chemical gardens (Huld et al. 2023).

### 4.4 (d) Bending and Tortuosity Distributions Differ Only Slightly Between Leptothrix Sheaths and Chemical Garden Tubes

Morphometry (the quantitative measurement of shapes) is a promising approach for differentiating biogenic and abiogenic structures (e.g., Hofmann et al. 2008; Rouillard et al. 2019). In paleontology, morphometry is already widely used for differentiating morphotaxa; such differences ought prima facie to be even more pronounced between organisms and non‐biological structures, and might therefore provide an objective way of discriminating between them, especially where large populations can be measured and compared statistically (Rouillard et al. 2019).

Here, the bending°/μm and tortuosity parameters were investigated following Hofmann et al. (2008), who compared microbial filaments with abiotic mineral fibres in order to assist the diagnosis of filamentous fabrics formed in subsurface cavities (“subsurface filamentous fabrics” or SFF). The microbial communities were diverse and unspecified; the abiotic fibres included “asbestiform fibrous minerals, whisker crystals of native silver and halotrichite, and fibers of volcanic glass”. Hofmann et al. (2008) found that the tortuosities of SFF, abiogenic mineral fibres and microbial filaments were similar. However, the bending values of SFF were high (median 2.5°/μm), resembling microbial filaments (median 0.87°/μm, with maximum values exceeding 10°/μm) more than abiotic mineral fibres (which were narrowly distributed around a median of 0.15°/μm and rarely exceeded 1.0°/μm). Hofmann et al. inferred that low bending values with narrow ranges are more typical of abiotic than with biotic filaments.

In our study, by contrast, the biogenic *Leptothrix* filaments showed a narrower range in both bending and tortuosity than the chemical gardens (and the moss agate), as evident from Figure 4. *Leptothrix* sheaths with thicker mineral coatings are typically straighter with lesser degrees of bending and tortuosity (Figure S8), whereas less mineralized “immature” filaments exhibit more sinuous shapes and tortuosity. This trend may be due in whole or part to the increased rigidity of the more mature tubules. Even if they were originally straight, less rigid “immature” filaments are easily bent when mechanically disturbed, for instance during pipetting onto slides (or during burial prior to fossilization). By contrast, more highly mineralized “mature” filaments will break rather than bend when disturbed. This trend would likewise apply to filaments experiencing mechanical stresses during the taphonomic process and may also be observed in the fossil record.

While our measurements do not cleanly discriminate *Leptothrix* from chemical gardens (or moss agates), they do show that filaments with a bending of < 0.5°/μm or a tortuosity > 2 are relatively unlikely to be *Leptothrix*. Morphometric analysis can be used to confirm or exclude certain proposed origins of mineralized tubules, but should be used as part of a holistic and contextual approach alongside other chemical and morphological indicators of biogenicity.

### 4.5 (e) Implications for the Diagnosis of Natural Samples

**FIGURE 7:**
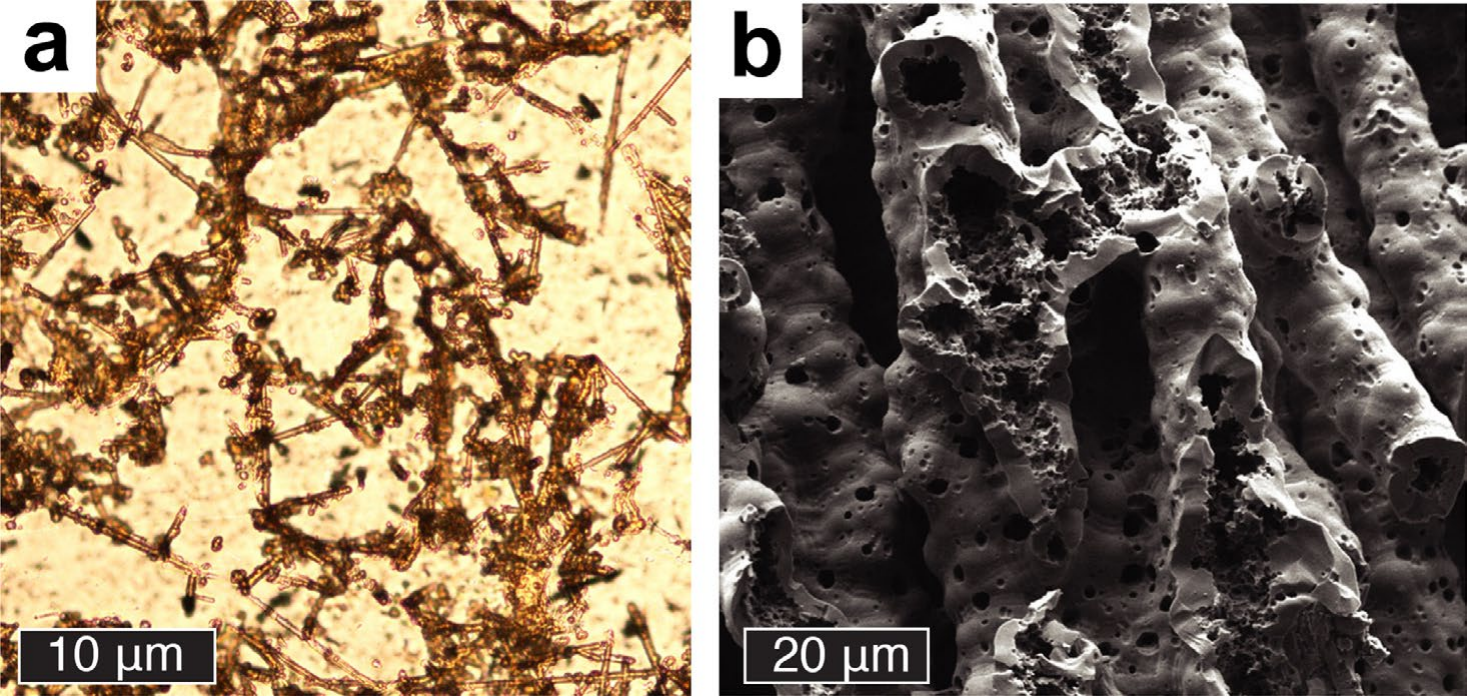
Micrographs showing microtextural features of naturally occurring iron (oxyhydr) oxide tubules from (a) Optical micrograph and (b) scanning electron micrographs of filaments in the Jan Mayen Si‐Fe mound (Johannessen et al. 2019).

We note that some of our results may shed light on the general palaeobiology of iron‐oxidizing bacteria. For example, a fossilized community of *Leptothrix* might be expected to have regions of straight tubes corresponding to more mature/auto‐mineralized colonies and regions of more sinuous, tortuous tubes corresponding to less mature/auto‐mineralized colonies (even though the latter, if secondarily encrusted by iron (oxyhydr)oxides, might be just as thick). The straight filaments (Figure 7a) observed by Johannessen et al. (2019) resemble mature *Leptothrix*, for example.

Chemical gardens can and do form in nature (Barge, Cardoso, et al. 2015). García‐Ruiz et al. (2017) and Kotopoulou et al. (2021) showed that natural waters from the Ney spring in California, which have been influenced by serpentinization, are sufficiently alkaline (pH 11.89) and silica‐rich (4326 ppm) to produce chemical garden tubules fundamentally similar to those reported here when suitable seed grains are introduced. Likewise, soluble transition metal sulfate and chloride salts are widespread in diverse natural settings—e.g., where pyrite weathering has occurred or in the vicinity of acid‐sulfate hot springs and fumaroles—and early oceans were Fe^2+^‐rich (Johnson et al. 2008). Thus, the microscopic chemical garden tubules prepared for this study may plausibly have natural counterparts, although it is a matter of debate whether those counterparts have already been found and misinterpreted as microfossils (e.g., McMahon 2019). Returning to the problem of how to determine the biogenicity of more ambiguous examples, the remainder of this section asks whether diagnostically useful textural and chemical differences between *Leptothrix* and chemical gardens might persist through diagenetic maturation or metamorphism.

We suggest that the microtextural differences have good preservation potential under some circumstances and can already be used to evaluate the biogenicity of some relatively young natural materials. Contrasting smooth interior and rough exterior surface textures are visible in tubules within the massive accumulations of iron oxide found at Cerro Colorado, Rio Tinto, Spain, which represent fossilized river terraces about two million years old (Figure 6a,b). Barge et al. (2016) tentatively suggest that these tubules might be field examples of chemical gardens. However, at least some of the observed varieties are certainly fossils formed by the encrustation of filamentous microorganisms and plants, as previously recognized by Fernández‐Remolar and Knoll (2008). This is evident from the petaloid cross‐section of the interior cavities of some tubes (compare Figure 6f,g). Consistent with our observations of the encrustation of *Leptothrix*, these fossils have smooth, well‐defined interior surfaces imprinted by the texture of the organism, and much rougher and more irregular outer surfaces. Mineralized microbial filaments in samples from inactive hydrothermal vents at the Main Endeavor Field, (north‐eastern Pacific Ocean) illustrate the same contrast (Li et al. 2017).

Natural chemical environments may favor continued precipitation of iron (oxyhydr)oxides as a secondary coating which may obscure original surface microtextures. Indeed, encrustation has thickened the filaments at Cerro Colorado more than tenfold (Figure 6a). A transition to low‐oxygen conditions or rapid encasement in a suitable medium could slow or halt secondary oxide mineralization in some environments (Stumm and Lee 1961; Butts 2014). Nevertheless, in the most ancient materials, recrystallization and chemical alteration are likely to have overprinted original microtextural features (Johannessen et al. 2019; Hirsch et al. 2025). Concentrically zoned simple filaments of hematite and talc found in Jurassic ophicalcite in Italy by McMahon et al. (2021), which resemble both microfossils and chemical gardens, are now too coarsely crystalline for the original texture to be reconstructed. Morphologically more impressive hematite filaments were described by Trewin and Knoll (1999) from early diagenetic calcite (CaCO_3_) veins in lacustrine limestones from the Scottish Middle Devonian. Trewin and Knoll interpreted these filaments as fossilized *Leptothrix*‐like IOB. The presence of a central cavity in many of the filaments supports this view (Figure 6c). However, in some of the filaments, this cavity has been narrowed, completely closed, or modified into an irregular suture by recrystallization of the iron oxide (Figure 6d,e). Such recrystallization lessens the utility of both surface microtexture and filament diameter for determining the biogenicity of filaments. Nevertheless, the inner surfaces, where they exist, are generally smoother than the outer surfaces, consistent with the biological interpretation advanced by Trewin and Knoll (Figure 6c).

Entombment by silica as opposed to carbonate may better preserve original textures of iron (oxyhydr)oxide filaments and tubules, especially if silica cementation is early and rapid (Hofmann et al. 2008). However, the controversial hematite filaments reported by Dodd et al. (2017) from Archaean/Palaeprotoerozoic chert/jasper in the Nuvvuagittuq greenstone belt are now too coarsely crystalline for original textures to be observed. It must also be noted that many tubular features in the rock record are diffusion bands produced around linear objects; the original structure may have been a solid filament and not a hollow tube. The apparent smoothness or coarseness of these bands cannot be taken to imply anything about the biogenicity of the original object.

Although the difference in iron redox state between chemical gardens and *Leptothrix* sheaths is interesting, its potential to preserve through time is limited. Iron minerals are strongly susceptible to diagenetic and metamorphic modification, including oxidation–reduction processes (Posth et al. 2014). In early diagenesis, iron (oxhydr)oxide minerals are prone to reductive dissolution, especially in the presence of sulfide or iron‐reducing bacteria (e.g., Canfield 1989). Metamorphic alteration of hematite (an Fe^3+^‐oxide) to magnetite (which contains Fe^3+^ and Fe^2+^) appears to have been pervasive in many ancient jaspers (Frost 1979; Grenne and Slack 2003) and can be promoted by coexisting reduced phases such as siderite and organic carbon (Posth et al. 2014). Equally, hematite and other ferric minerals can form via the abiotic or microbially mediated oxidation of ferrous precursors, whether as an early diagenetic phenomenon, an alteration effect of oxidizing groundwater, or through recent weathering (e.g., Brown et al. 1997; Tarhan et al. 2018). In the famous filamentous microfossils of the 1.9 Ga Gunflint Formation of Canada, hematite occurs as a late‐stage replacement of ferrous aluminum silicates, and its occurrence therefore does not imply that these organisms were iron oxidizing bacteria (Wacey et al. 2021). Indeed, it has been suggested that much of the hematite occurring in Precambrian rocks (particularly in banded iron formations) occurs as a postdepositional replacement of ferrous silicates (Rasmussen et al. 2016).

## 5 Conclusion

Though the overall size, shape, and composition are similar in many ways, there are morphological and chemical differences between biomineralized biogenic *Leptothrix* tubules and abiogenic chemical garden tubules. Although these differences are useful in diagnosing the biogenicity of relatively young and unaltered iron mineral tubules in natural samples (and particularly for excluding the chemical garden hypothesis), they are likely to become increasingly obscured with the passage of time.

## Conflicts of Interest

Rights Retention Statement: For the purpose of open access, the author has applied a Creative Commons Attribution (CC BY) licence to any Author Accepted Manuscript version arising from this submission.

## Supporting information


Appendix S1.

## Data Availability

The data that support the findings of this study are available from the corresponding author upon reasonable request.
