# Supplementary material for: Troubles With Tubules: How Do Iron‐Mineral Chemical Gardens Differ From Iron‐Mineralized Sheaths of Iron Oxidizing Bacteria?
Source: Geobiology. 2025 May 14;23(3):e70021. doi: 10.1111/gbi.70021 (PMC12078188; doi:10.1111/gbi.70021)
Supplement: Supplementary file 1 — Appendix S1. [file GBI-23-e70021-s001.docx]

Supplementary material

Troubles with tubules: how do iron-mineral chemical gardens differ from iron-mineralized sheaths of iron oxidizing bacteria?

## **Contents**

**Supplementary Methods**

Leptothrix culturing

Fluorescence staining

Electron microscopy

**Supplementary Figures**

**Fig S1** - A petrographic thin section of Devonian basalt-hosted moss agate from Campsie, Scotland

**Fig S2** - A petrographic thin section of calcite-veined lacustrine limestone from the Devonian deposits near Fochabers, NE Scotland (Tynet Burn fish bed)

**Fig S3** - *Leptothrix* sp. FB and SP6 in transmitted light micrographs

**Fig. S4** - Examples of coalescence of *Leptothrix* sp. (FB) filaments into bundles*.*

**Fig. S5** *-* Scanning electron micrographs of “immature” *Leptothrix* sheaths.

**Fig. S6 -** Transmission electron micrographs of *Leptothrix* sheaths (a-d) and chemical gardens (e&f)

**Fig. S7** - Transmitted electron micrographs of highly mineralized sheaths of *Leptothrix*.

**Fig. S8** - Morphometric comparison of maturing *Leptothrix*.

**Fig. S9 -** Powder XRD diffraction pattern of chemical garden material

**Fig. S10 -** *Leptothrix* sp. FB in transmitted light micrographs overlay with fluorescence micrographs

**Supplementary Tables**

**Table S1** - Diameters and statistical values for Leptothrix and chemical gardens.

**Table S2** - Mössbauer analysis data.

## **Supplementary Methods**

### ***Leptothrix culturing***

*Leptothrix* cultures were grown at ambient temperature in the dark in Angelova isolation medium (I.M.) (Angelova et al., 2015). (pH 7.0) containing glucose (C_6_H_12_O_6_) 0.150 g, (NH_4_)_2_SO_4_ 0.500 g, Ca(NO_3_)_2_ 0.010 g, K_2_HPO_4_ 0.050 g, MgSO_4_·7H_2_O 0.050 g, KCl 0.050 g, CaCO_3_ 0.100 g, cyanocobalamin (vitamin B12) 0.00001 g, and thiamine (vitamin B1) 0.0004 g per 1,000 ml of distilled water (Angelova et al, 2015). As an iron source 10% w/v iron filings with a diameter of 250–350 µm (“mesh 40”) were added.

### ***Fluorescence Staining***

SYBR Gold was used to detect living intact cells in *Leptothrix* cultures. SYBR Gold is a nucleic acid stain with fluorescence excitation maxima of ≈ 300 nm and ≈ 495 nm, and fluorescence emission maximum of ≈ 537 nm (Tuma et al. 1999). Frozen 20x SYBR Gold solution was stored in Eppendorf tubes and thawed immediately prior to use. A 1:1000 dilution of SYBR Gold stain was created 10 µl of diluted stain was added per 100 µl of cell culture for a final dilution of 1:10 000. Individual flocculates of IOB were removed from medium with micropipette and placed in 2 mL Eppendorf tube and mixed with fully diluted SYBR Gold at a 1:1 ratio. The stained culture was observed on Leica DM 4000 B microscope using fluorescence excitation filter cube K3 (470-490 nm).

***Transmission Electron microscopy (TEM)***

For TEM observations experimental samples were fixed in 3% glutaraldehyde in 0.1 M sodium cacodylate buffer, pH 7.3, for 2 hours then washed in three 10 minute changes of 0.1 M sodium cacodylate. Specimens were then post-fixed in 1% osmium tetroxide in 0.1 M sodium cacodylate for 45 minutes, then washed in three 10 minute changes of 0.1 M sodium cacodylate buffer. These samples were then dehydrated in 50%, 70%, 90% and 100% ethanol (X3) for 15 minutes each, then in two 10-minute changes in propylene oxide. Samples were then embedded in TAAB 812 resin. Sections of 1 μm thickness were cut on a Leica Ultracut ultramicrotome, stained with toluidine blue, and viewed with a light microscope to select suitable areas for investigation. Ultrathin sections of 60 nm thickness were cut from selected areas, stained in uranyl acetate and lead citrate then viewed in a JEOL JEM-1400 Plus TEM. Images were collected on a GATAN OneView camera.

## **Supplementary Figures**


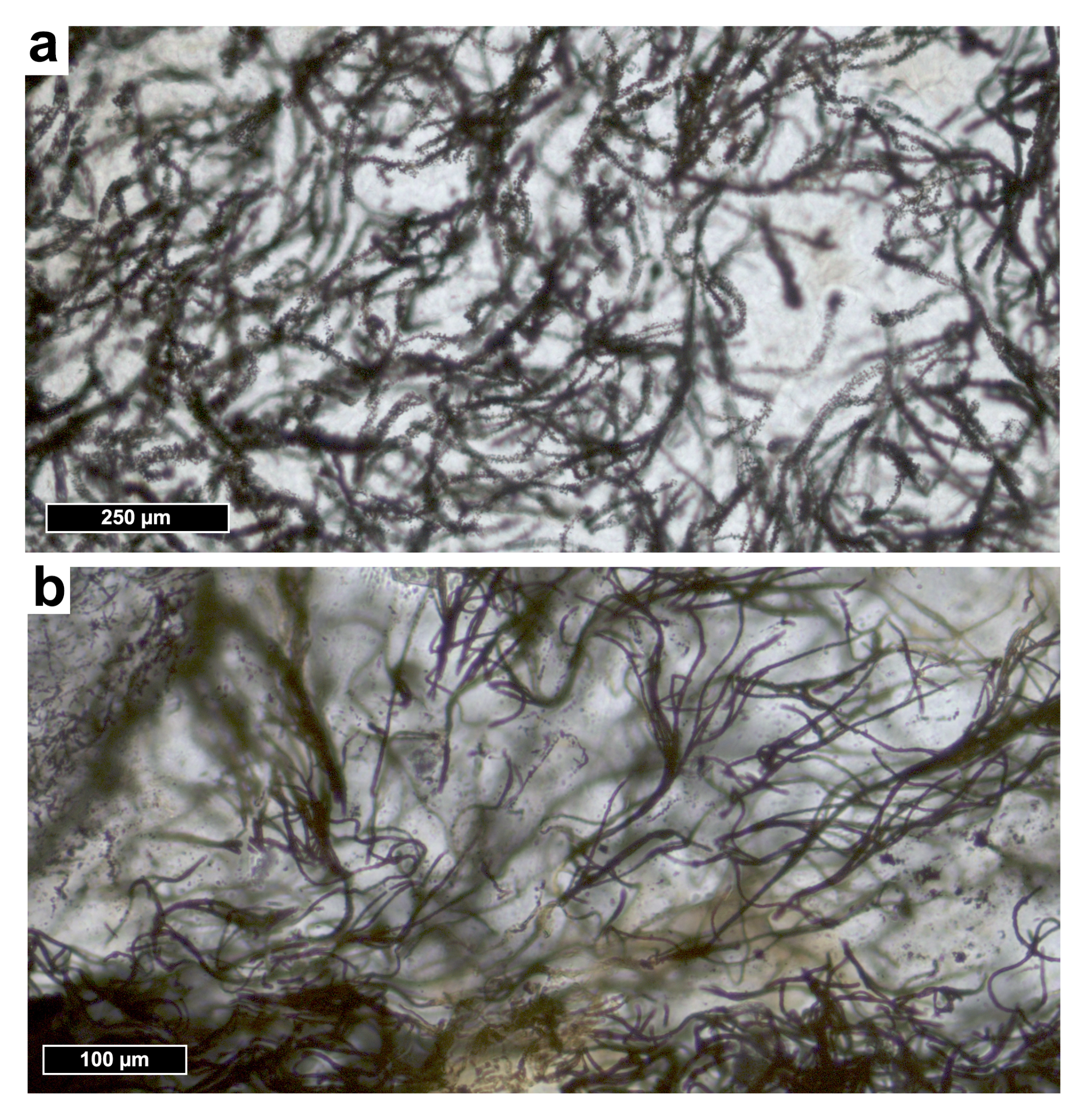
**Figure S1. A petrographic thin section of Devonian basalt-hosted moss agate from Campsie, Scotland.** **(a&b)** Micrographs of moss agates containing filamentous fabrics with typical filament tortuosity and bending observed in these samples.


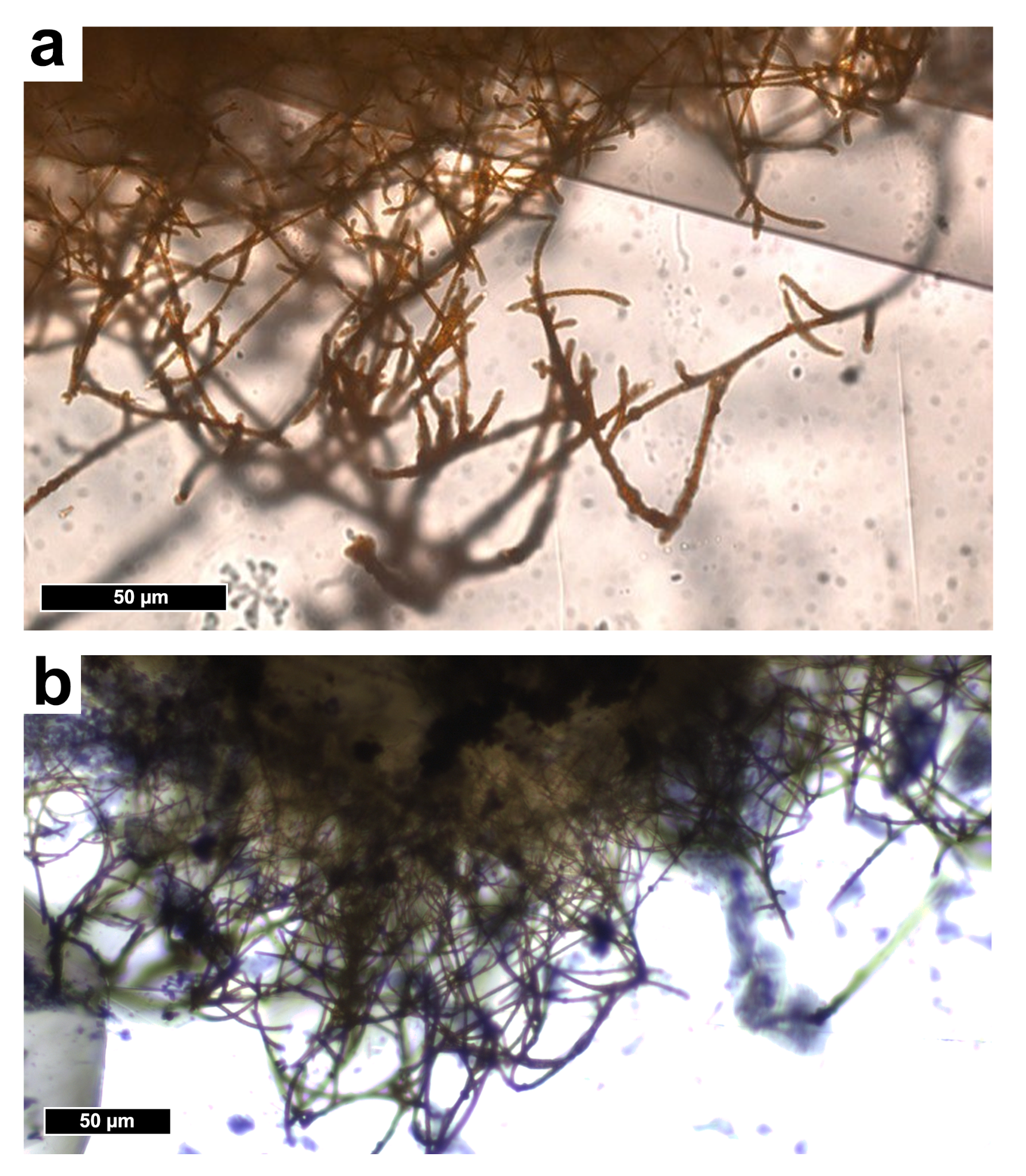
**Figure S2. A petrographic thin section of calcite-veined lacustrine limestone from the Devonian deposits near Fochabers, NE Scotland (Tynet Burn fish bed).** Micrographs of calcite vein showing filamentous networks of iron oxide.


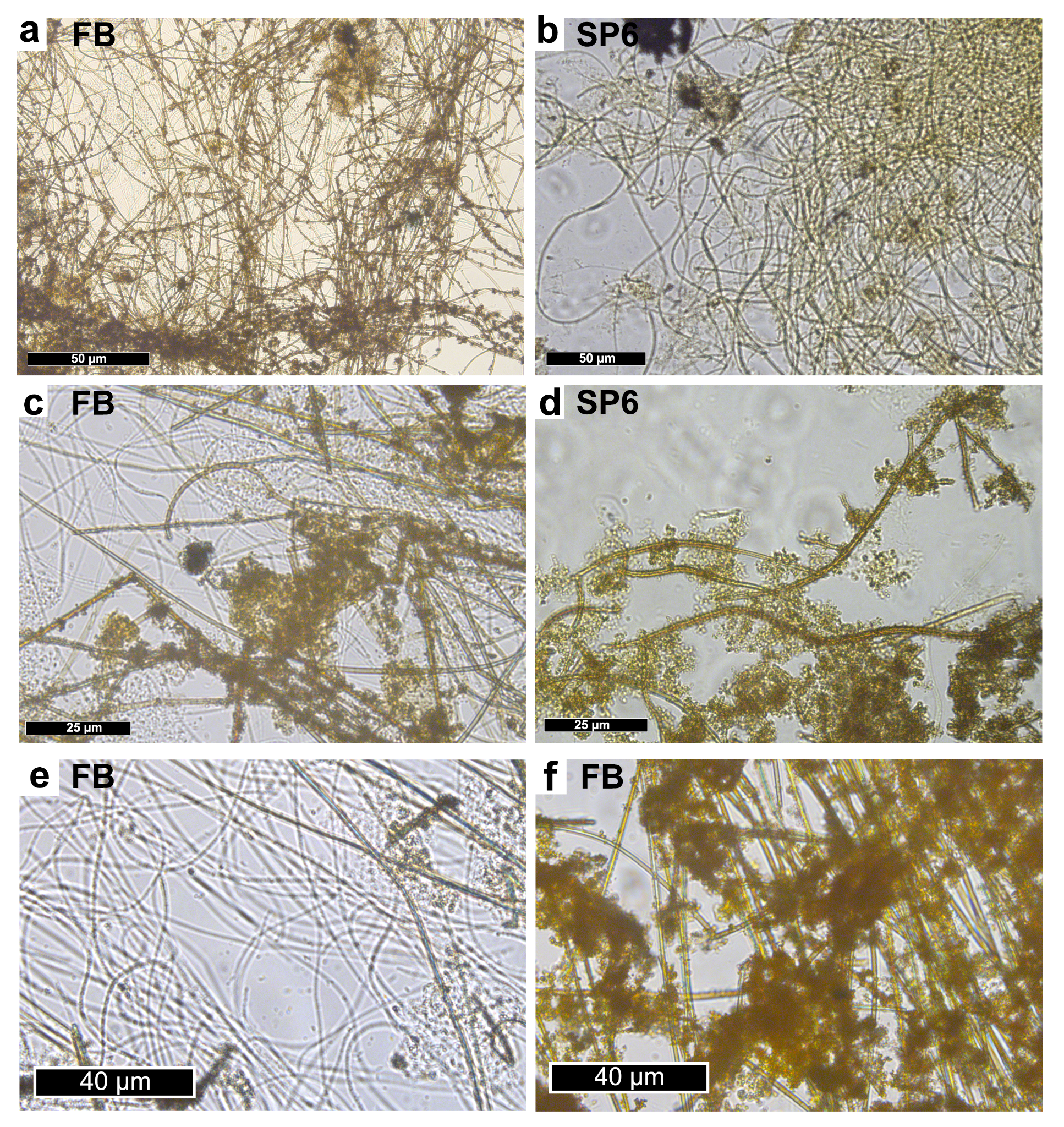


**Figure S3. *Leptothrix* sp. FB and SP6 in transmitted light micrographs.**, Semitransparent, “immature” sheathed filaments with little or no mineralization **(e)**, cultures with a mixture of immature and more mature highly mineralised filaments **(a, b, c, & d)**, and even more “mature” sheathed filaments with highly mineralization and straighter, more brittle morphologies **(f)**.


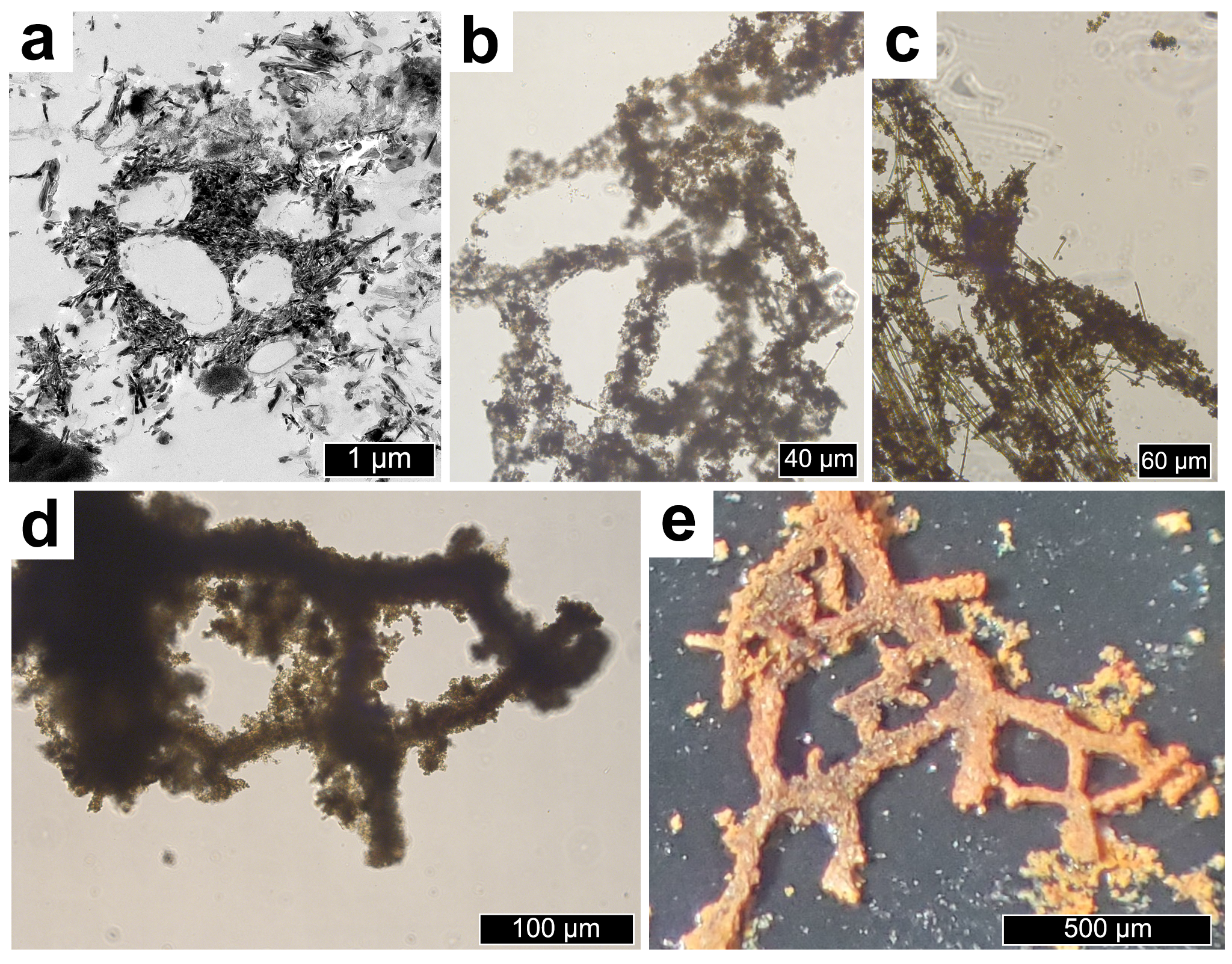


### **Figure S4. Examples of coalescence of *Leptothrix* sp. (FB) filaments into bundles**. **(a)** Transmitted electron micrograph showing transverse section through four Leptothrix filaments bundled together. **(b)** Transmitted light micrograph showing coalescence structure in 43-day-old culture. **(c)** Coalescence structure in freshly inoculated Figgate Burn culture. **(d, e)** Transmitted and reflected light micrographs showing a larger macroscopic filament aggregate with a high degree of mineralization.


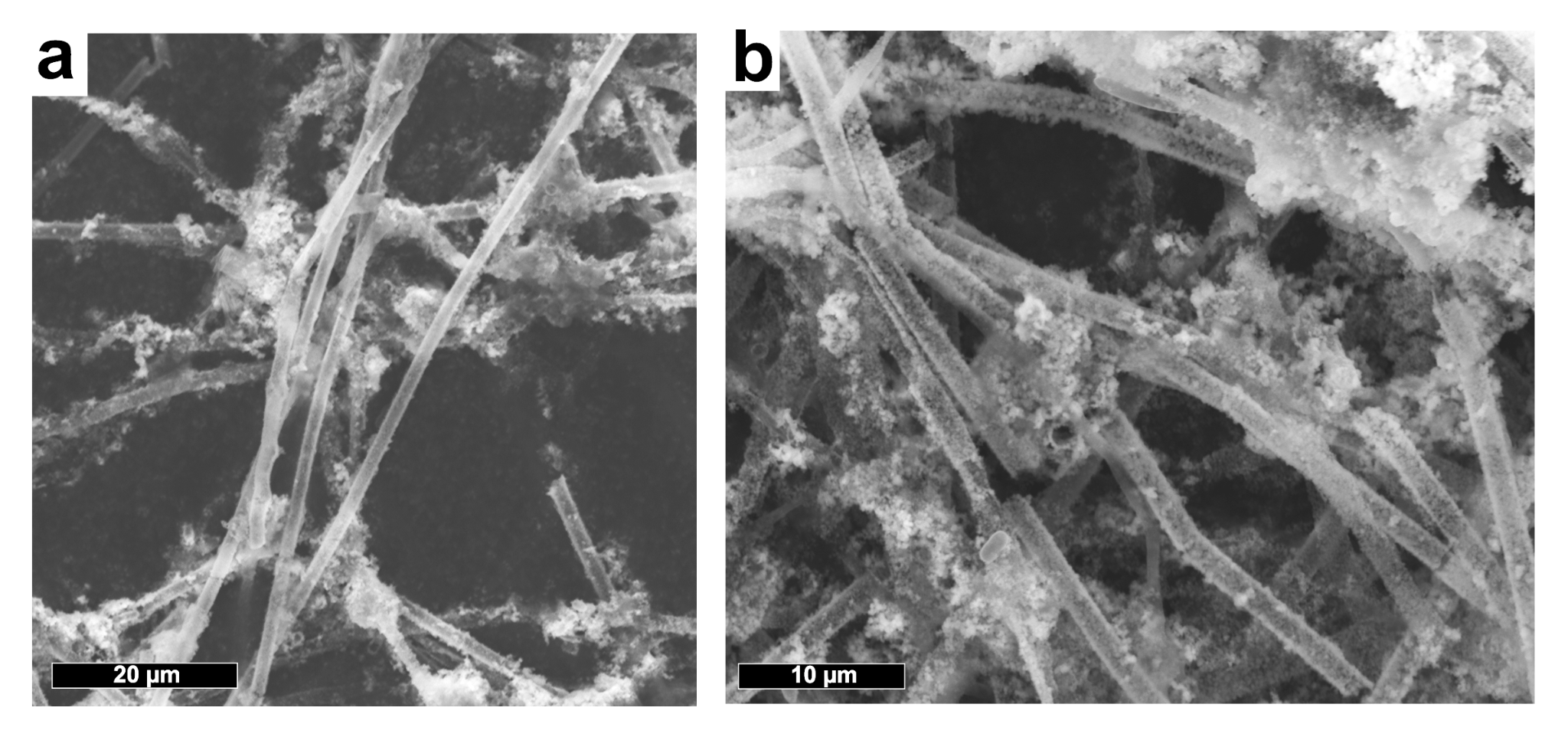
**Figure S5: Scanning electron micrographs of “immature” *Leptothrix* sheaths.** **(a&b)** The sheaths in this sample have not accumulated secondary mineralization, appearing smooth and somewhat transparent.


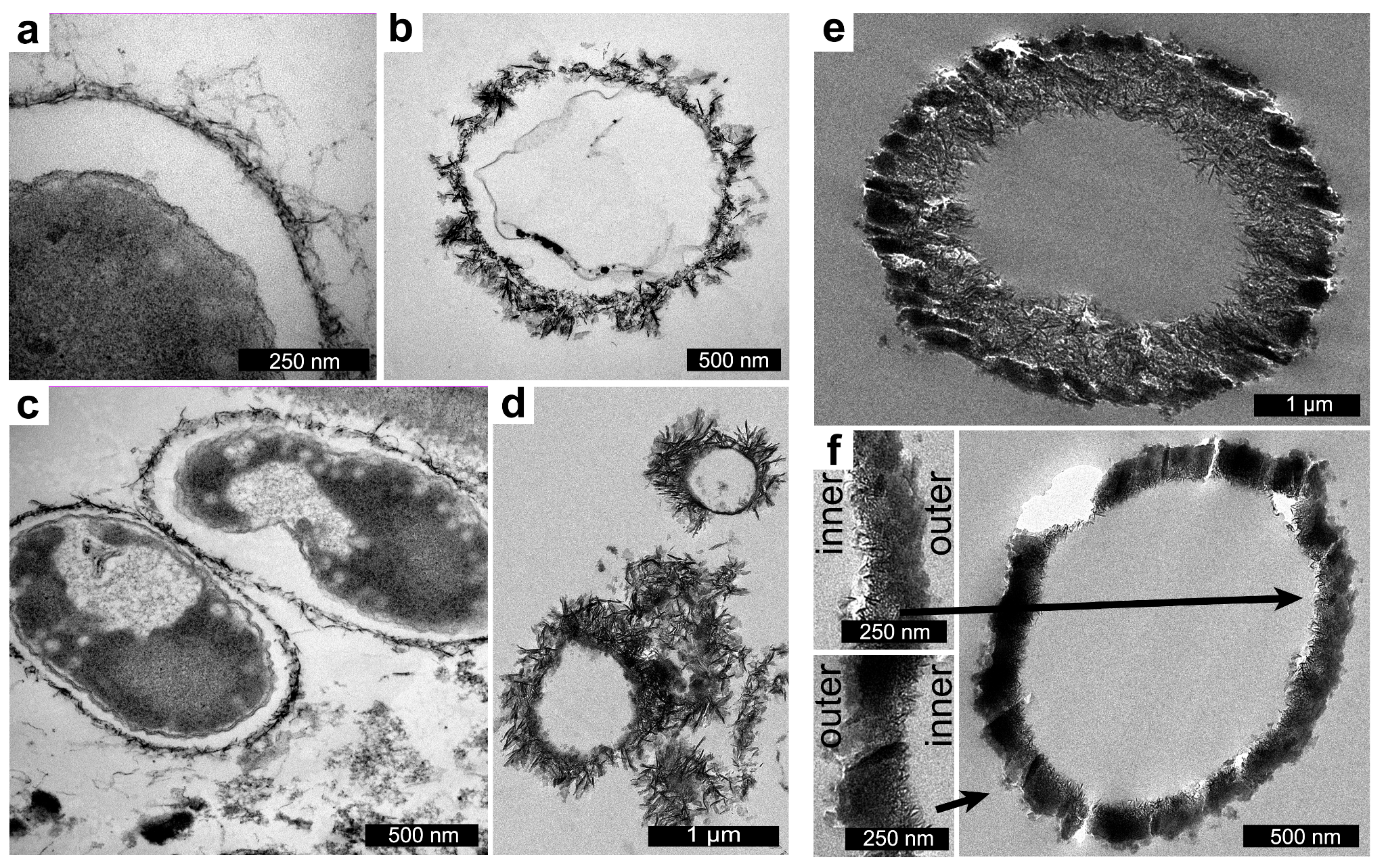


**Figure S6. Transmission electron micrographs of *Leptothrix* sheaths (a-d) and chemical gardens (e&f).** Biogenic filaments are all ~1 µm in diameter **(a-d)** whereas chemical garden tubules have more variable diameters **(e&f)**. Rough and diffuse exterior surface textures and smoother inner surfaces are observed on the biogenic tubules **(a-d)**, while this textural difference is reversed on chemical garden tubules **(e&f)**. While occasionally chemical garden tubes may initially appear dense and smooth on both inner and outer surfaces **(f)** closer inspection reveals contrasting textural differences even in these examples (see insets).


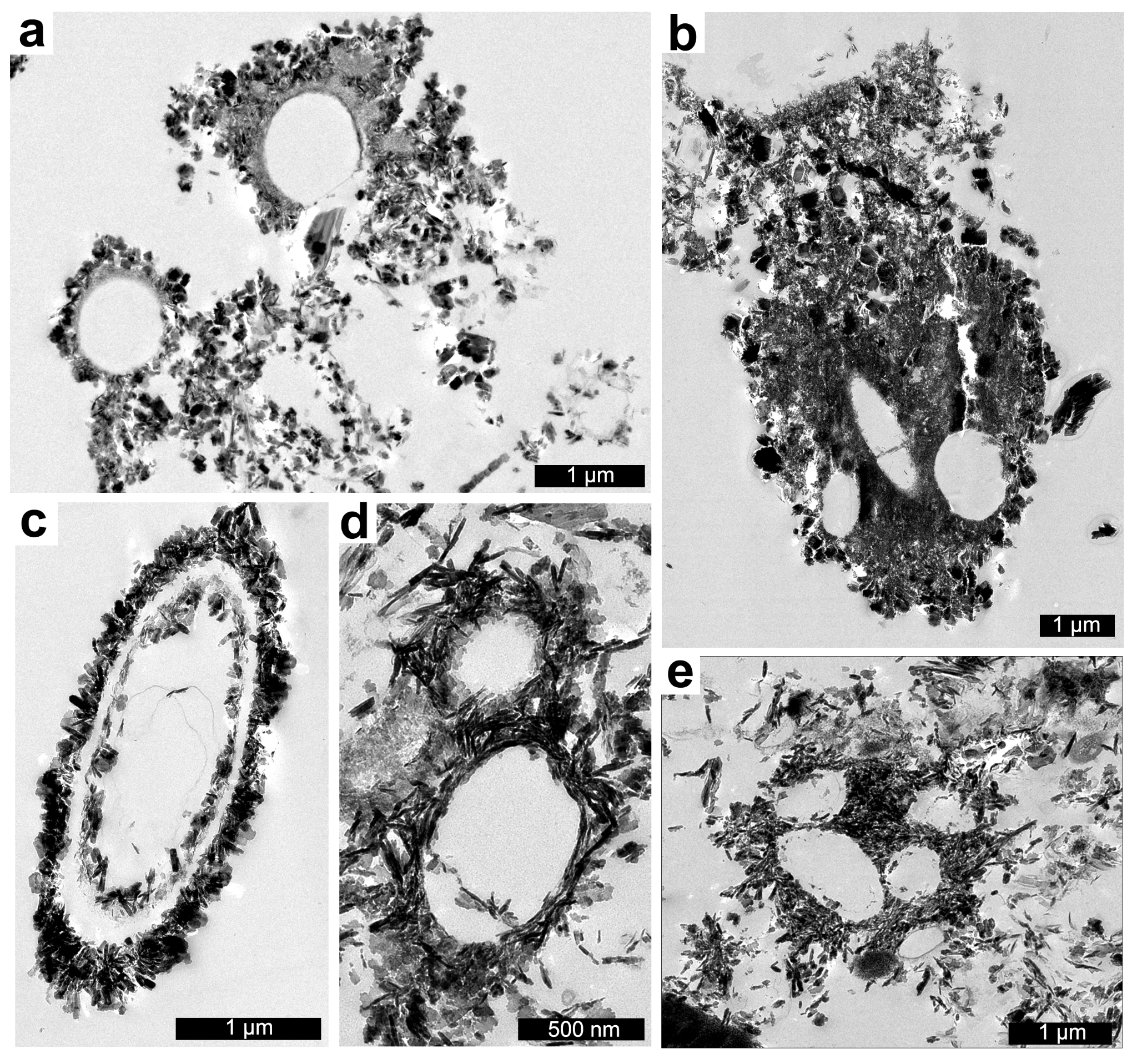
**Figure S7. Transmitted electron micrographs of highly mineralized sheaths of *Leptothrix*.** Rough and diffuse textures observed on the exterior surfaces of biogenic tubules from a 93 day old culture of *Leptothrix* sp. FB. These highly mineralised sheath exteriors display diverse granular (**a&b**) and platy (**d**) textures. In one case the mineralization forms two distinct and seperate layers **(c)**. Extensive mineralization can lead to coalescence of multiple filaments into multi-filament structures (see also Fig S4)

### **
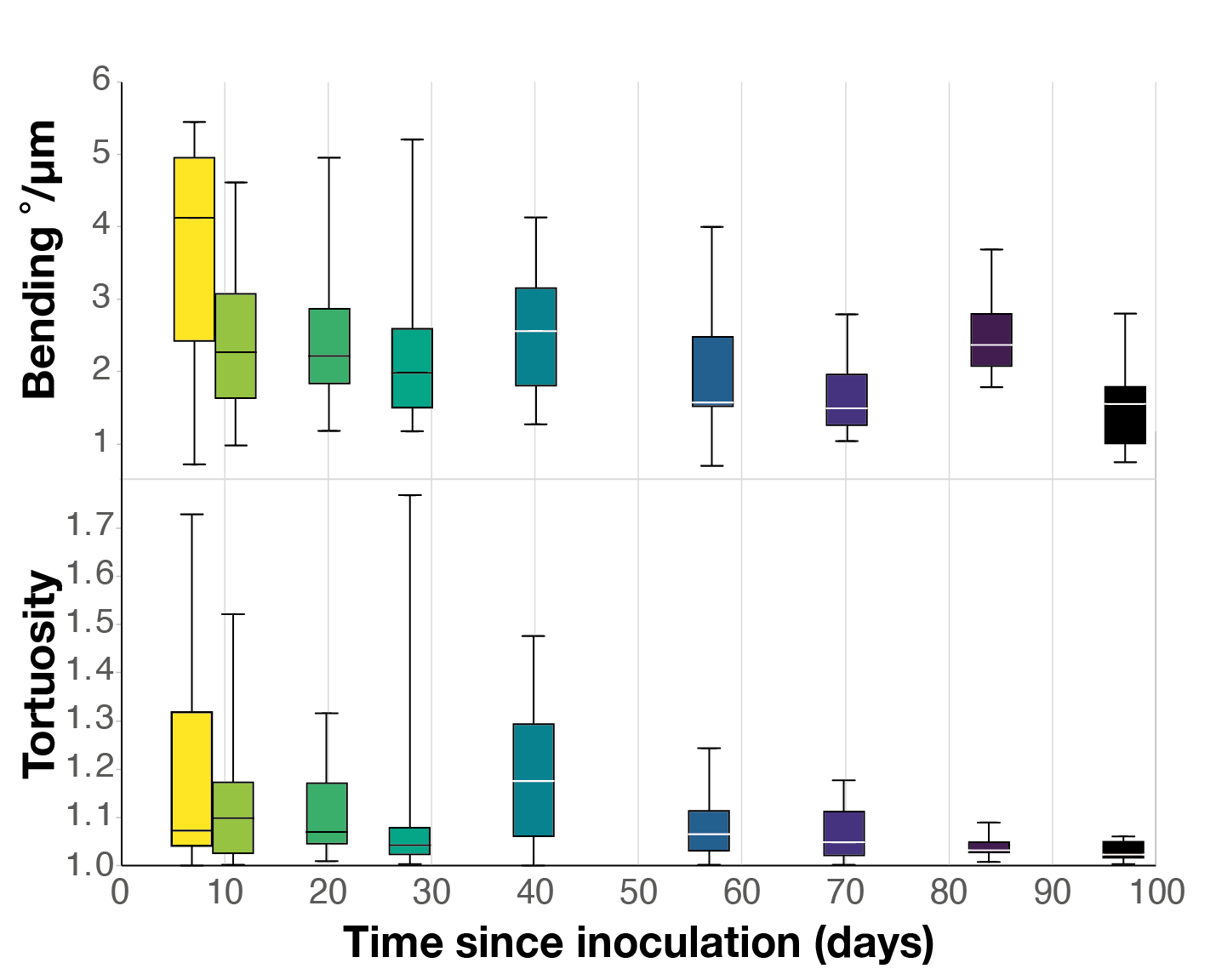
**

### **Figure S8. Morphometric comparison of maturing *Leptothrix*.** Box-and-whisker plot showing that tortuosity and degrees of bending per µm tend to decrease on average and become more homogeneous as populations of *Leptothrix cholodnii* SP-6 bacteria age over 97 days.


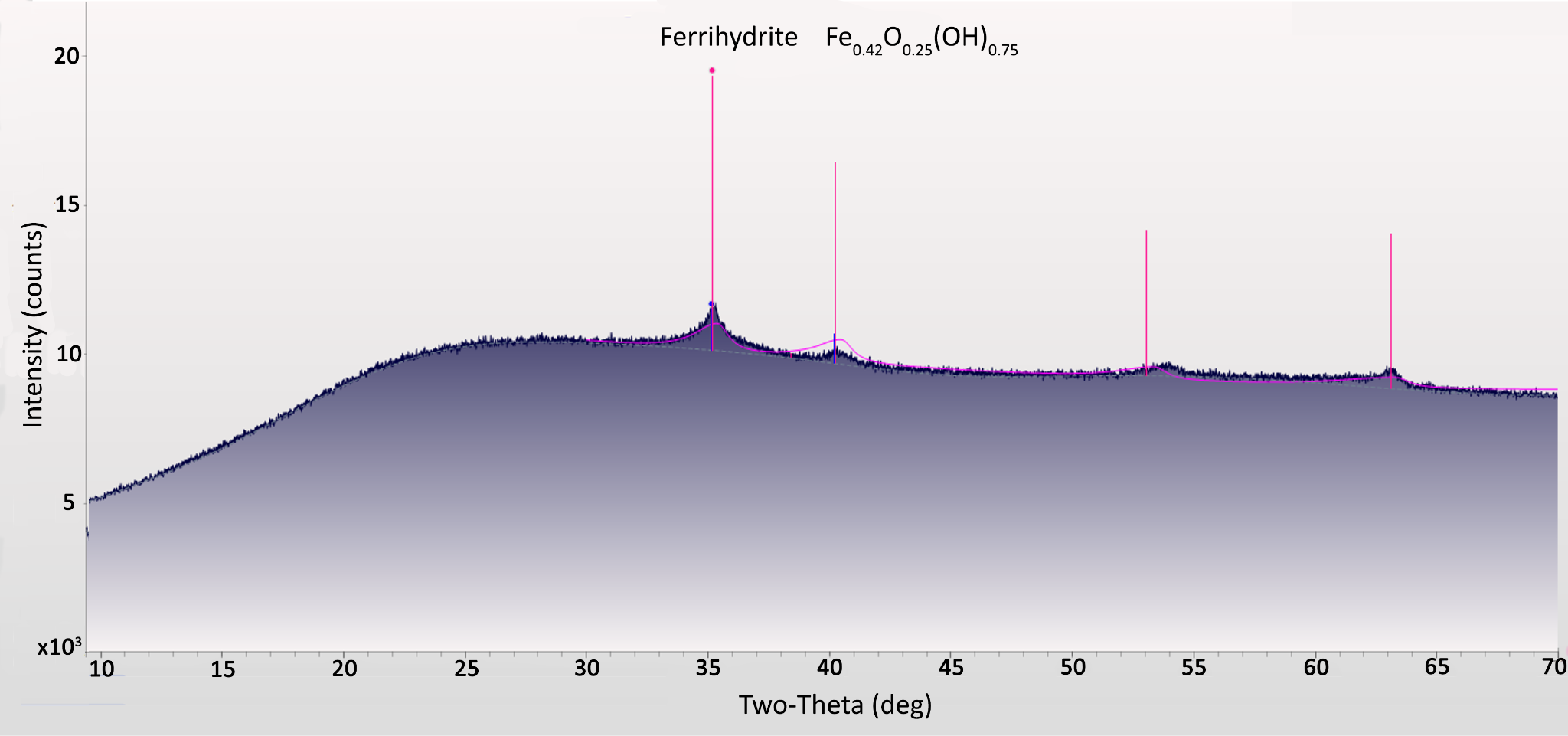
**Figure S9. Powder XRD diffraction pattern of chemical garden material.** Diffraction pattern shows chemical garden samples have predominantly amorphous composition with a few distinct peaks consistent with ferrihydrite (indicated by pink lines).


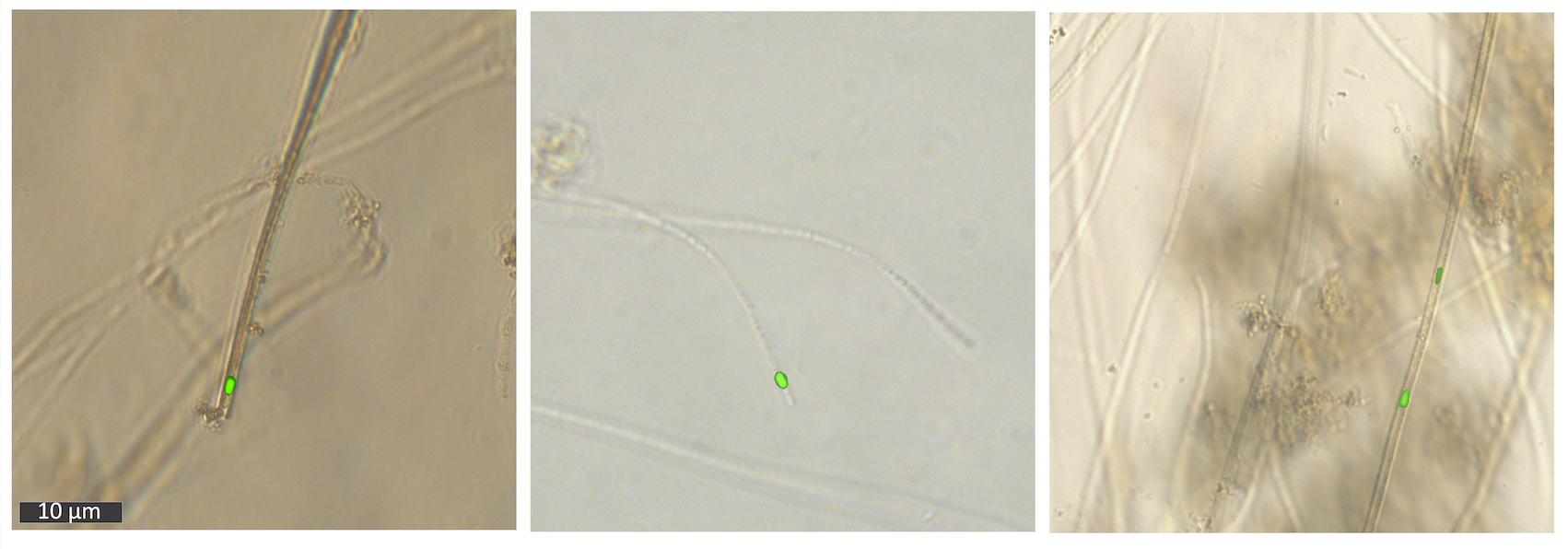


**Figure S10. *Leptothrix* sp. FB in transmitted light micrographs overlay with fluorescence micrographs.** “Immature” Leptothrix sheaths with little or no mineralization visible. Sheaths are mostly devoid of living cells (green) stained with SYBR Gold.

## **Supplementary Tables**

|  | **Leptothrix** | **Chemical Gardens** |
| --- | --- | --- |
|  | **External Diameter** | |
| **Min (µm)** | **0.8** | **1.7** |
| **Max (µm)** | **1.7** | **11.8** |
| **Mean (µm)** | **1.1** | **4.2** |
| **Median (µm)** | **1.1** | **3.7** |
| **n** | **98** | **92** |
|  | **Internal Diameter** | |
| **Min (µm)** | **0.74** | **2.2** |
| **Max (µm)** | **1.17** | **11.5** |
| **Mean (µm)** | **0.87** | **5.4** |
| **n** | **10** | **11*** |
|  | **Bending** | |
| **Min (µm)** | **0.7** | **0.1** |
| **Max (µm)** | **5.4** | **5.8** |
| **Mean (µm)** | **2.3** | **1.5** |
| **Median (µm)** | **2** | **0.8** |
| **n** | **102** | **30** |
|  | **Tortuosity** | |
| **Min (µm)** | **1.0** | **1.0** |
| **Max (µm)** | **1.8** | **2.7** |
| **Mean (µm)** | **1.1** | **1.2** |
| **Median (µm)** | **1.1** | **1.0** |
| **n** | **102** | **30** |

### **Table S1. Morphometric properties of mineralized *Leptothrix* sheaths and chemical garden tubes.** **Unrepresentative subset:* Internal diameter data was gathered from scanning electron micrographs. Internal diameters could only be measured when tubules were broken (and aligned correctly with the detector). Chemical garden tubule diameters are quite variable and breaks tend to appear in wider tubes in preference to narrow tubes, reflected in the wider average internal tubule diameter.

| **Sample** | **Centre shift** | **DQ** | **Full-width  half-maximum** | **Area (%)** | **Assignment** |
| --- | --- | --- | --- | --- | --- |
| ***Leptothrix*** | **0.37** | **0.53** | **0.28** | **100** | **Fe^3+^** |
| ***Carbonate chemical*** | **0.37** | **0.52** | **0.29** | **39.8** | **Fe^3+^** |
| ***garden*** | **0.37** | **0.83** | **0.47** | **56.2** | **Fe^3+^** |
|  | **1.26** | **3.2** | **0.31** | **4.1** | **Fe^2+^** |
| ***Silicate chemical*** | **0.38** | **0.57** | **0.32** | **44.9** | **Fe^3+^** |
| ***garden*** | **0.39** | **0.95** | **0.39** | **35.3** | **Fe^3+^** |
|  | **1.27** | **3.24** | **0.28** | **19.8** | **Fe^2+^** |

**Table S2.** Table showing the CS, DQ, FWHM and Area (%) of the analysed samples and where "CS" represents centroid shift (the midpoint of a doublet), "DQ" is quadrupole splitting (the distance between the left and right peaks in a doublet), "FWHM"= full width at half maximum, and "Area" represent the absorption fraction for each doublet (corresponding to the fraction of Fe).
